# Supplementary material for: Biochemical and structural insights into SARS-CoV-2 polyprotein processing by Mpro
Source: Sci Adv. 2022 Dec 9;8(49):eadd2191. doi: 10.1126/sciadv.add2191 (PMC9733933; doi:10.1126/sciadv.add2191)
Supplement: Supplementary file 1 — Supplementary Text Figs. S1 to S15 Tables S1 to S4 [file sciadv.add2191_sm.pdf]

Science Advances
AAAS

Supplementary Materials for

Biochemical and structural insights into SARS-CoV-2 polyprotein processing by Mpro

Ruchi Yadav *et al.*

Corresponding author: Eddy Arnold, arnold@cabm.rutgers.edu; Patrick R. Griffin, pgriffin@scripps.edu; Francesc X. Ruiz, xavier@cabm.rutgers.edu

Sci. Adv. **8**, eadd2191 (2022)
DOI: 10.1126/sciadv.add2191

The PDF file includes:

Supplementary Text
Figs. S1 to S15
Tables S1 to S4
References

Other Supplementary Material for this manuscript includes the following:

Data S1 and S2

Supplementary Text

Materials and Methods

Gel LC-MS/MS

Samples (gel bands) were in-gel digested with sequencing grade trypsin (ThermoFisher) using standard protocols (*85, 86*) and analyzed by nano-LC-MS/MS.

Nano-liquid chromatography-tandem mass spectrometry (LC-MS/MS)

Samples were analyzed using a Q Exactive HF tandem mass spectrometer coupled to a Dionex Ultimate 3000 RLSCnano System (Thermo Scientific). Samples were loaded onto a fused silica trap column Acclaim PepMap 100, 75umx2cm (ThermoFisher). After washing for 5 min at 5 µl/min with 0.1% TFA, the trap column was brought in-line with an analytical column (Nanoease MZ peptide BEH C18, 130A, 1.7um, 75 um x 250 mm, Waters) for LC-MS/MS. Peptides were eluted using a segmented linear gradient from 4 to 90% B (A: 0.2% formic acid, B: 0.08% formic acid, 80% ACN): 4–15% B in 5 min, 15-50% B in 50 min, and 50-90% B in 15 min. Mass spectrometric data were acquired using a data-dependent acquisition procedure with a cyclic series of a full scan from 250-2000 with resolution of 120,000 followed by MS/MS (HCD, relative collision energy 27%) of the 20 most intense ions (charge +1 to +6) and a dynamic exclusion duration of 20 sec. Major peptides were manually confirmed.

Database Search

The peak lists of the LC-MSMS were generated by Thermo Proteome Discoverer (v.2.1) into MASCOT Generic Format (MGF) and searched against custom supplied sequences plus a database composed of common lab contaminants using in house version of X!Tandem (*87*). Search parameters were as follows: fragment mass error: 20 ppm, parent mass error: +/- 7 ppm; fixed modification: none; flexible modifications: oxidation on methionine for the primary search, and dioxidation of methionine and glutamine to pyro-glutamine at the refinement stage; protease specificity: tryptic allowing 2 miscleavages for the primary search and semitryptic allowing 5 miscleavages at the refinement stage. Only spectra with loge <-2 were included in the final report. Peptides belonging to target proteins were manually inspected to determine exact processed sequences.

Integrative structural modeling of nsp7-8 using I-TASSER

For the structural predictions of the nsp7-8 polyprotein, a similar integrative modeling approach to nsp7-11 modeling was employed using the I-TASSER server.

For nsp7-8 modeling, we employed a similar two-run approach using the amino acid sequence and varying the experimental input parameters. Run 1 included using PDB:6YHU as a template, along with distance constraints from crosslinking MS (XL-MS), and secondary structural restraints for nsp7 and nsp8 from solved X-ray crystal structures to generate Models 1-5 **(Fig. S10B)**. Run 2 also used PDB:6YHU as a template, distance constraints from crosslinking MS (XL-MS), and secondary structural restraints for nsp8 from the crystal structures to generate Models 6-10 **(Fig. S10B)**. We employed the secondary structural elements from PDB: 6YHU as the HDX-MS solvent exchange profile of the individual proteins is similar to the nsp7-8 polyprotein **(Fig. 3A).** These data suggest that the individual proteins and polyprotein contain similar secondary structural elements. The 10 output models were assessed against the experimental (i) HDX-MS, (ii) XL-MS, and the (iii) SAXS data, similar to what we did for nsp7-11 polyprotein.

As the HDX-MS profile of the nsp7-8 polyprotein resembles the profile of nsp7-8 within the nsp7-11 polyprotein **(Fig. 3A)**, we used models of nsp7-8 as a structural template for the structural modeling of nsp7-11 **(Fig. 3B)**. Models 5 and 6 were selected as templates for nsp7-11 modeling based on their agreement with the XL-MS and HDX-MS data. Model 5 is most consistent with the distance restraints obtained by XL-MS, whereas Model 6 is most consistent with the solvent exchange profiles obtained by HDX-MS. As such, we wanted to limit experimental bias and maximize the conformational variety for the integrative modeling of nsp7-11.

The nsp7-8 integrative structures have been deposited in the PDB-Dev databank under accession codes PDBDEV_00000119. They are also provided in the SM as PyMOL sessions.

Results

Integrative structural modeling of SARS-CoV-2 nsp7-8 polyprotein

Similarly, as with nsp7-11, we first used analytical SEC coupled to a multi-angle light scattering (MALS) and SAXS detection (SEC-MALS-SAXS) to analyze the in-solution assembly state and structural features of the nsp7-8 polyprotein. Two major peaks were detected from the

SEC-MALS analysis of the nsp7-8 polyprotein with the main peak corresponding to an equilibrium between monomeric and dimeric states **(Fig. S9A).** SAXS analysis was conducted for both the oligomeric states by using evolving factor analysis (EFA) to separate the scattering of the monomer and dimer components **(Fig. S9B)**. A linear Guinier plot for both the states suggested the presence of stable protein samples with no aggregation **(Fig. S9C)**. A bell-shaped (Gaussian) curve at lower q values was observed in the Kratky plot indicating that the sample contains folded domains with no significant disorder **(Fig. S9D)**. The pair-distance distribution function, P(r), which is related to the shape of the sample, indicated a globular shaped protein for both the monomeric and dimeric forms **(Fig. S9E)**. The Rg and the Dmax values calculated from the P(r) are: 33.7 Å and 118 Å for the dimer, and 25.2 Å and 88 Å for the monomeric nsp7-8 **(Table S2)**.

The I-TASSER workflow generated an ensemble of ten nsp7-8 models **(Fig. S10B)**. All models show the characteristic nsp7 helical bundle with a more variable conformation of nsp8. Unlike nsp7-11, the nsp7-8 models don't adopt the "golf-club" conformation in the nsp8 domain. Analysis of the nsp7-8 polyprotein models suggest that the nsp8 domain is more compact in the context of this polyprotein intermediate. Visual inspection of models in nsp7-11 Group A suggest that this extended helical conformation is stabilized by nsp7 on one side and by nsp9 and nsp10 from the other. Thus, in the case of nsp7-8, the lack of scaffolding by the absent nsp9-11 domains may disfavor the extended helical conformation. This absence of extended nsp8 helix in the context of nsp7-8 might enhance nsp7-8 polyprotein stability, limiting its ability to sample dynamic conformations and ultimately making it less amenable to cleavage by Mpro.

All the models satisfied most of the crosslinks with distances equal to or less than 30 Å, the theoretical upper limit distance for the DSSO crosslinker **(Fig. S10C)**, of Model 7 able to satisfy all of the crosslinks. Moreover, all crosslinks are satisfied across all the models.

Next, we mapped the percent deuterium uptake at 10 s incubation in deuterated buffer on the models to assess the agreement of the modeled secondary structure elements to the in-solution dynamics of the polyprotein **(Fig. S10D)**. Using HDXer software we calculated deuterated fractions for peptide segments corresponding to the experimental data as a function of the experimental deuterium exposure times. We then plotted the computationally derived 10 s percent deuterium value for each model versus the experimentally determined 10 s percent deuterium value and calculated the RMSE. We observed similar agreement of all models with the experimental HDX-MS data, with only ~0.1 RMSE difference between Model 7, which shows highest RMSE

(least agreement), and Model 1, which shows the lowest RMSE (best agreement). Interestingly, the average RMSE for the individual domains was greater for nsp7 compared to nsp8, suggesting that the models do a better job at modeling nsp8. Model 6 was the only model where nsp7 had better agreement with HDX-MS experimental data compared to nsp8. For this reason, Model 6 was chosen as one of the template models for the nsp7-11 modeling.

Next, we reconstructed a three-dimensional shape (bead model) for the monomeric nsp7-8. Based on evaluation of the bead models **(Table S2)**, the reconstruction seemed somewhat unstable, which may be caused by conformational flexibility in solution. Nonetheless, all the models fitted well in the envelope **(Fig. S10E)**. The theoretical scattering profile of each monomer model was calculated using the *CRYSOL* software package and fitted against the experimental scattering profile **(Fig. S10F)**. The calculated scattering profiles fit well to the experimental scattering profile, as seen with the low χ^2 values. The calculated radius of gyration (R_g) values for the models were also comparable with the experimental R_g value of the monomeric nsp7-8 polyprotein (experimental R_g is 25.2 Å) calculated from the P(r) function, suggesting that the overall size of the model matches with the actual size of the polyprotein in solution.

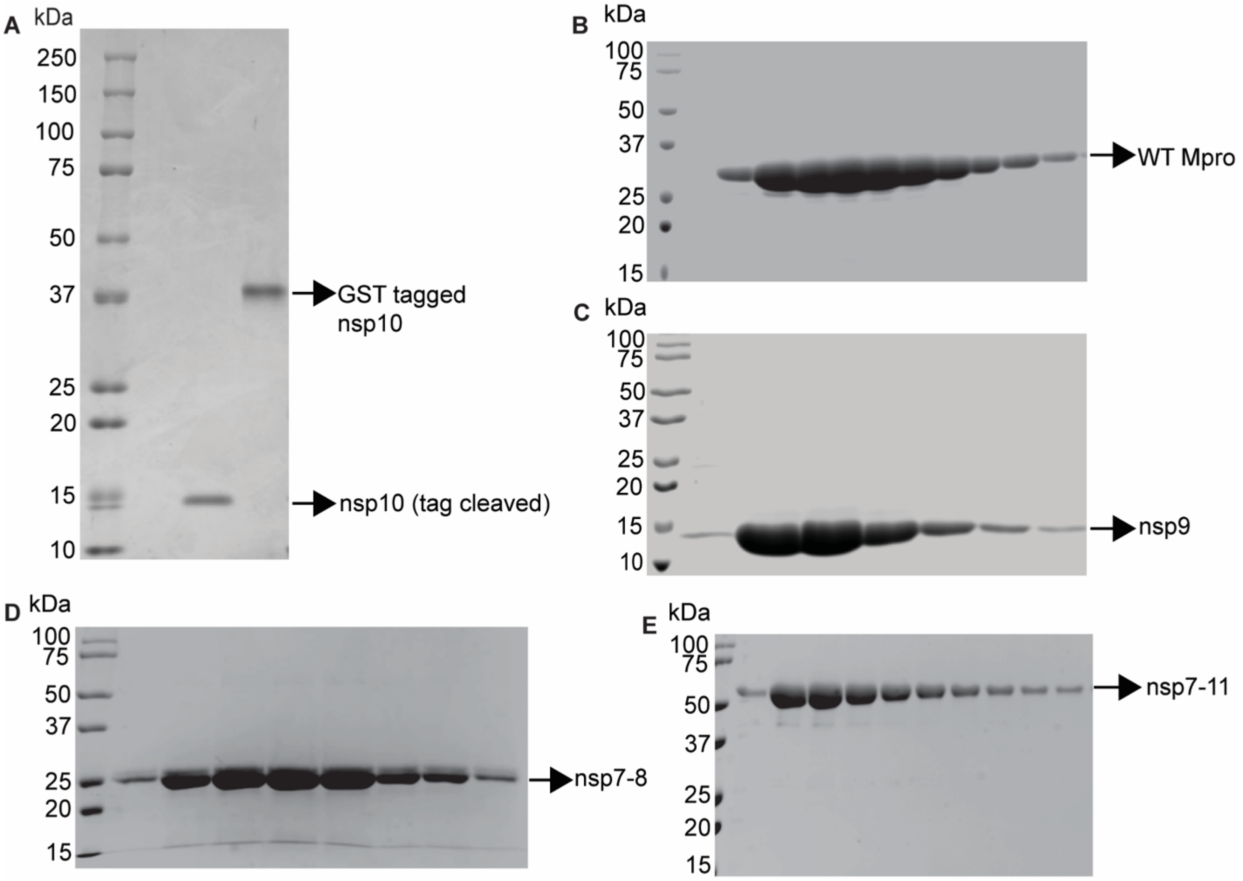

Fig. S1. Characterization of purified SARS-CoV-2 protein samples. SDS-PAGE of purified (A) nsp10, (B) WT Mpro, (C) nsp9, (D) nsp7-8, and (E) nsp7-11.

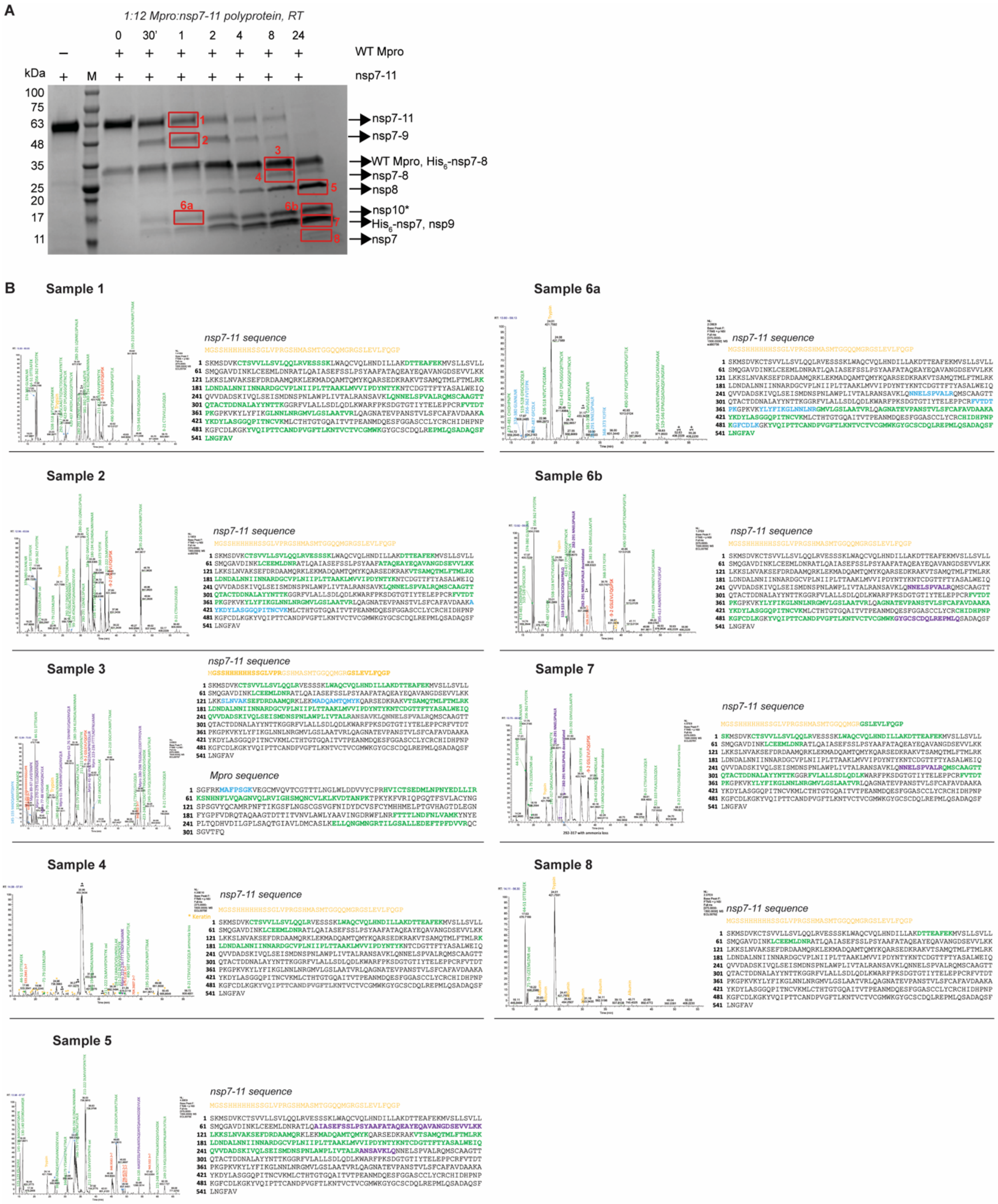
A
1:12 Mpro:nsp7-11 polyprotein, RT
0 30' 1 2 4 8 24
WT Mpro
nsp7-11
kDa
M
100
75
63
48
35
25
20
17
11
nsp7-11
nsp7-9
WT Mpro, His6-nsp7-8
nsp7-8
nsp8
nsp10*
His6-nsp7, nsp9
nsp7
B
Sample 1
Sample 2
Sample 3
Sample 4
Sample 5
Sample 6a
Sample 6b
Sample 7
Sample 8
nsp7-11 sequence
Mpro sequence

Fig. S2. Validation of nsp7-11 *in vitro* processing by WT Mpro. (A) SDS-PAGE of nsp7-11 processed by WT Mpro with representative protein bands confirmed by LC-MS/MS marked in red. *After 30 mins of incubation, the gel band corresponds to nsp10-11 polyprotein intermediate and after 24 h of incubation, the band corresponds to nsp10. The molecular mass of nsp11 is only 1.3 kDa so it couldn't be resolved on the SDS-PAGE gel. (B) Sample traces of peptide IDs in LC-MS/MS analysis of the marked protein bands. The peptides in green and blue are the tryptic peptides identified from the abundant peaks and low intensity peaks respectively, while the ones in purple are non-tryptic peptides that were cleaved by trypsin on one terminus and Mpro on the other terminus. The sequence in yellow represents the tag.

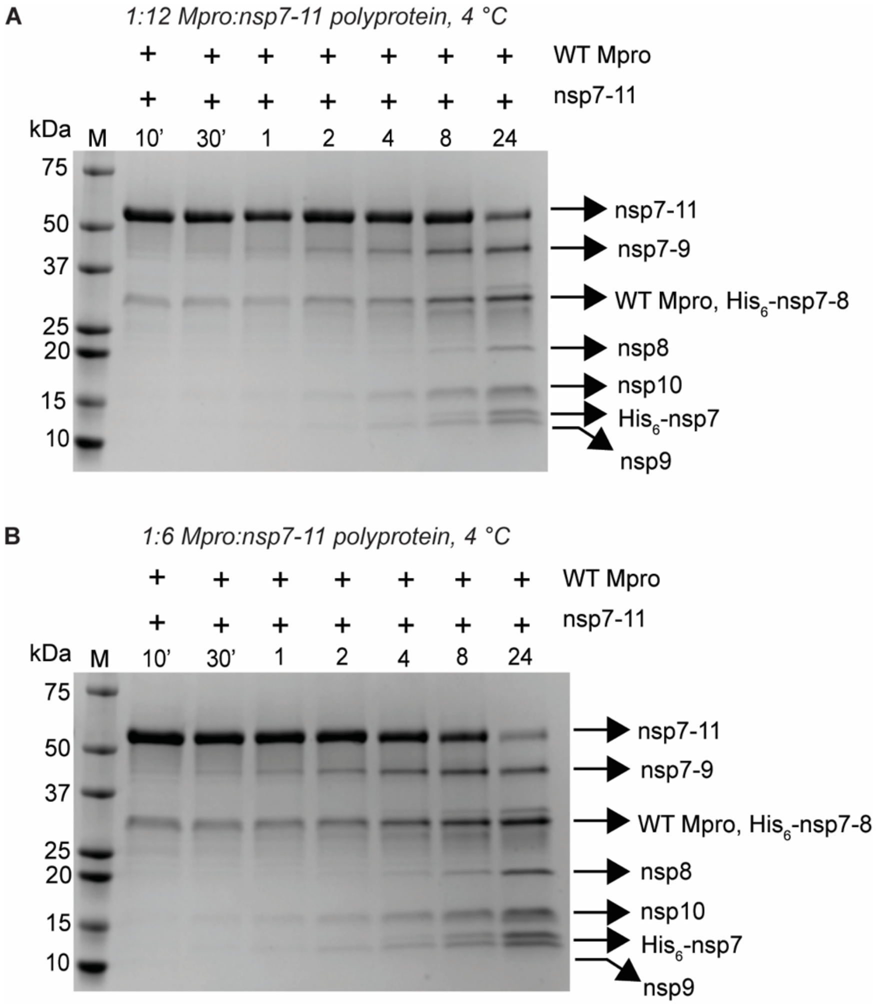

Fig. S3. *In vitro* analysis of SARS-CoV-2 nsp7-11 polyprotein processing by WT Mpro at 4 °C using different enzyme-substrate ratios. (A) SDS-PAGE of nsp7-11 processed by WT Mpro at using a 1:12 Mpro:nsp7-11 polyprotein ratio. (B) SDS-PAGE of nsp7-11 processed by WT Mpro using a 1:6 Mpro:nsp7-11 polyprotein ratio.

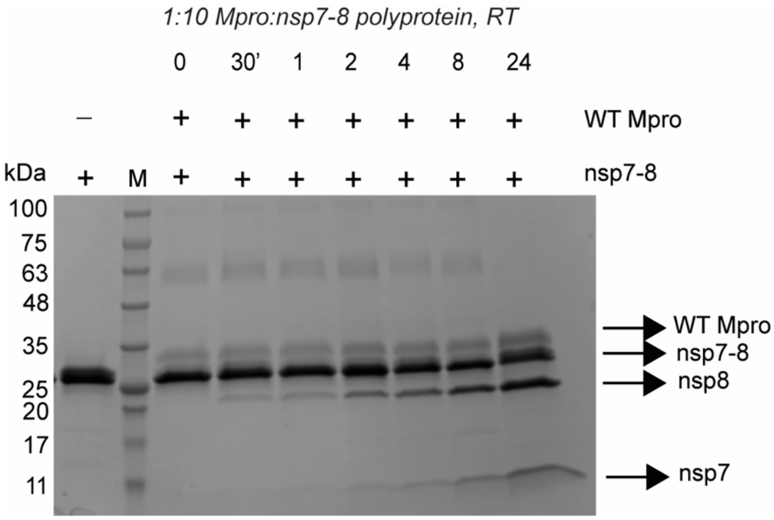

Fig. S4. ***In vitro*** **analysis of nsp7-8 polyprotein processing by WT Mpro.** SDS-PAGE of nsp7-8 processed by WT Mpro at ambient temperature (~25 °C).

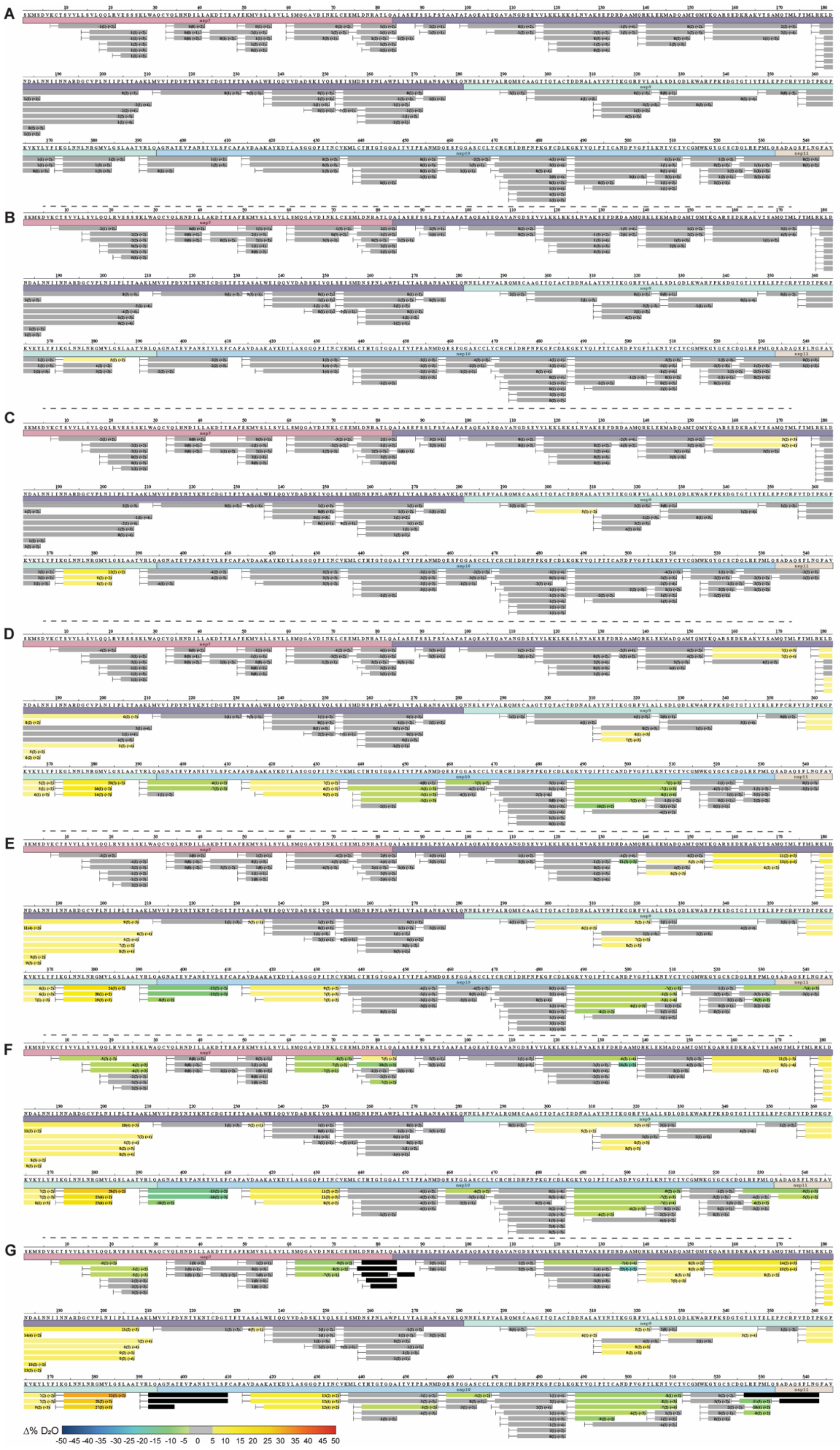
A
B
C
D
E
F
G
Δ% D2O
-50 -45 -40 -35 -30 -25 -20 -15 -10 -5 0 5 10 15 20 25 30 35 40 45 50

Fig. S5. Pulsed HDX-MS of SARS-CoV-2 nsp7-11 with WT Mpro. Sequence coverage of differential pulsed HDX-MS analysis of nsp7-11 versus nsp7-11 with WT Mpro after (A) 600 s, (B) 1800 s, (C) 3600 s, (D) 7200 s, (E) 14400 s, (F) 28800 s, and (G) 86400 s of cleavage reaction time. Color scale represents changes in deuterium uptake (30 s incubation in deuterated buffer) over the course of the cleavage reaction, with gray representing no significant change in deuterium uptake and black representing peptides no longer identifiable in the mass spectrometer.

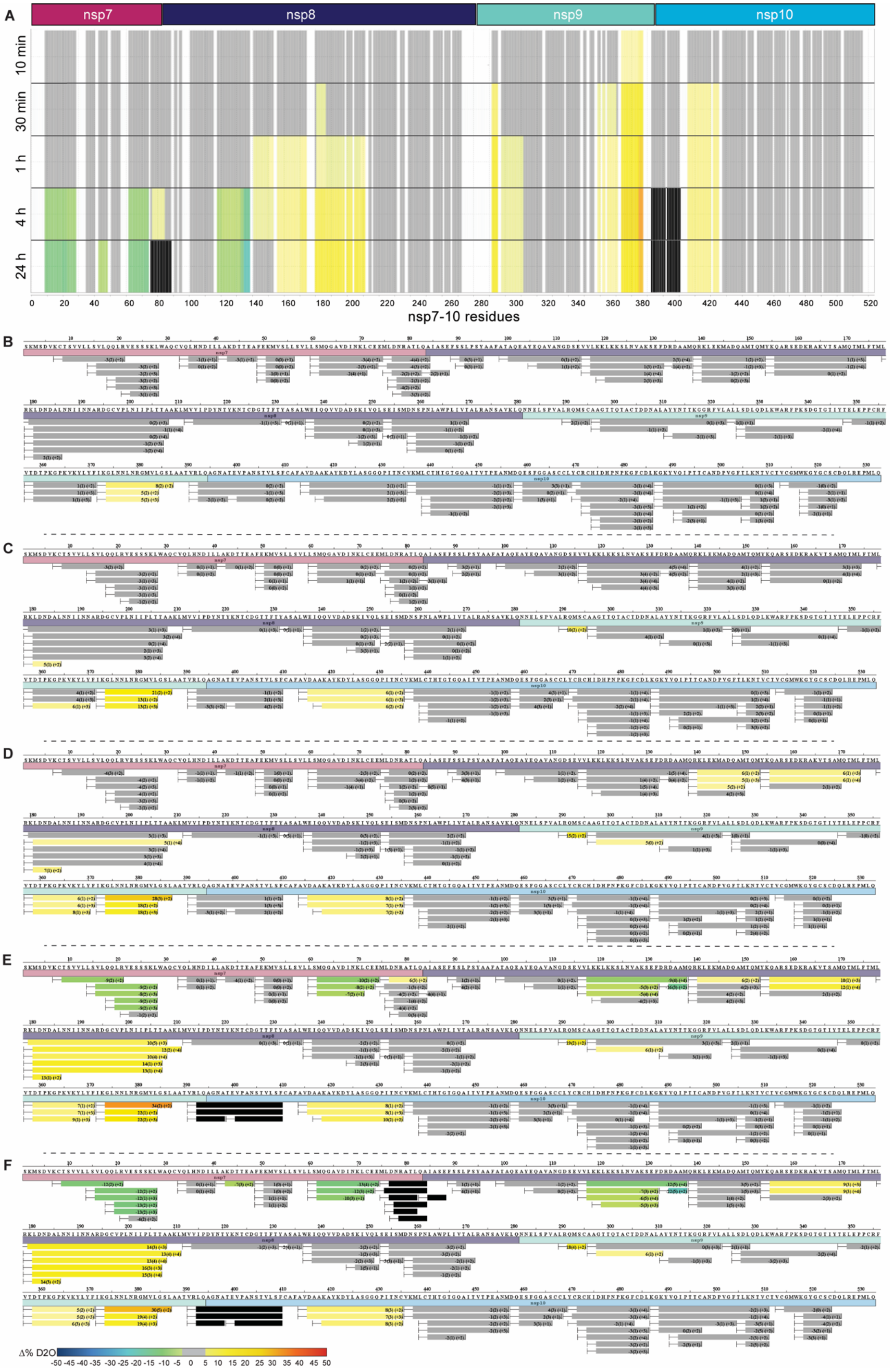
A
nsp7
nsp8
nsp9
nsp10
10 min
30 min
1 h
4 h
24 h
nsp7-10 residues
B
C
D
E
F
Δ% D2O

Fig S6. Pulsed HDX-MS of SARS-CoV-2 nsp7-10 with WT Mpro. (A) Pulsed HDX-MS analysis of nsp7-10 with WT Mpro. Sequence coverage of differential pulsed HDX-MS analysis of nsp7-11 versus nsp7-11 with WT Mpro after (B) 600 s, (C) 1800 s, (D) 3600 s, (E) 14400 s, and (F) 86400 s of cleavage reaction time. Color scale represents changes in deuterium uptake (30 s incubation in deuterated buffer) over the course of the cleavage reaction, with gray representing no significant change in deuterium uptake, white denoting no sequence coverage, and black representing peptides no longer identifiable in the mass spectrometer.

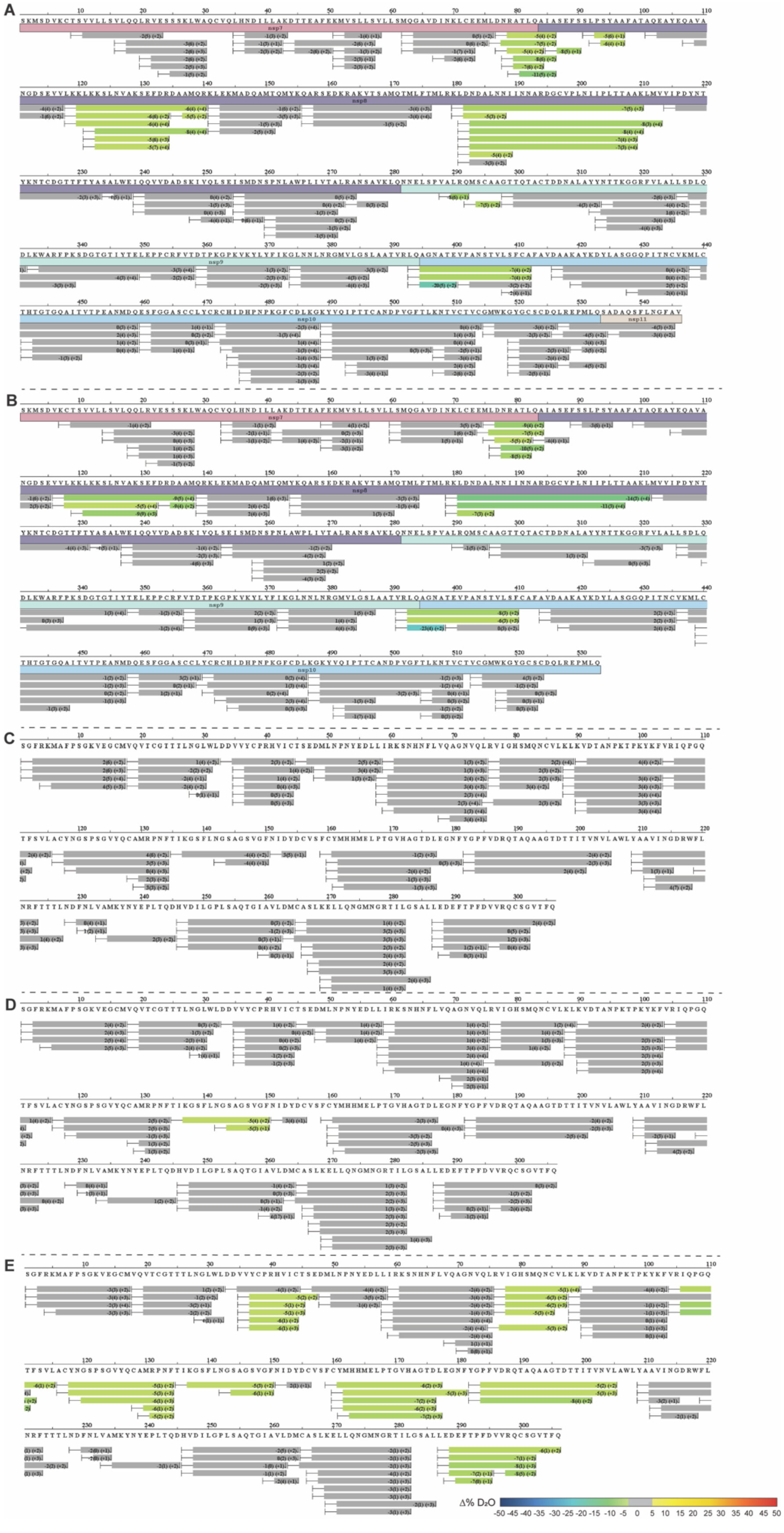
A
B
C
D
E
Δ% D2O
-50 -45 -40 -35 -30 -25 -20 -15 -10 -5 0 5 10 15 20 25 30 35 40 45 50

Fig. S7. HDX-MS of C145A Mpro complex with nsp7-10. Sequence coverage of differential HDX-MS analysis of (A) nsp7-11 versus nsp7-11 with C145A Mpro, (B) nsp7-10 versus nsp7-10 with C145A Mpro, (C) C145A Mpro versus C145A Mpro with nsp7-11 at 10 s, 60 s, 5 min, 30 min, and 1 h incubation in deuterated buffer, (D) C145A Mpro versus C145A Mpro with nsp7-11 at 10 s, 60 s, 5 min, 30 min, 1 h, and 12 h incubation in deuterated buffer and (E) C145A Mpro versus C145A Mpro with nsp7-11 at 12 h incubation in deuterated buffer only. Color scale represents changes in deuterium uptake, with gray representing no significant change in deuterium uptake.

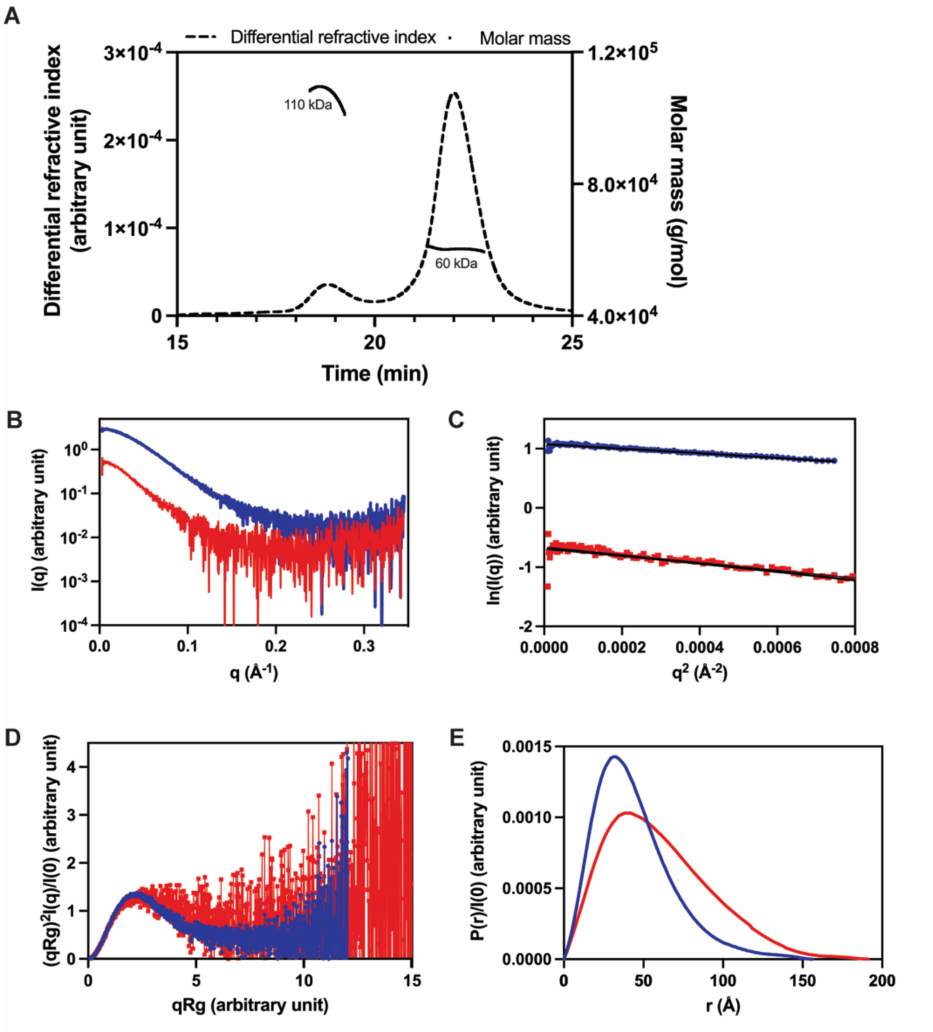

Fig. S8. SEC-MALS-SAXS analysis of SARS-CoV-2 nsp7-11 polyprotein. A) SEC-MALS chromatogram of nsp7-11 polyprotein. Dashed line represents the DRI (differential refractive index) signal, and the plain line represents the molecular weight of the corresponding peaks. B) SAXS scattering profiles with I(q) versus q as log-linear plots, C) Guinier fit, D) Dimensionless Kratky plots and E) Pair-wise distribution curve of monomeric (blue) and dimeric (red) nsp7-11 polyprotein.

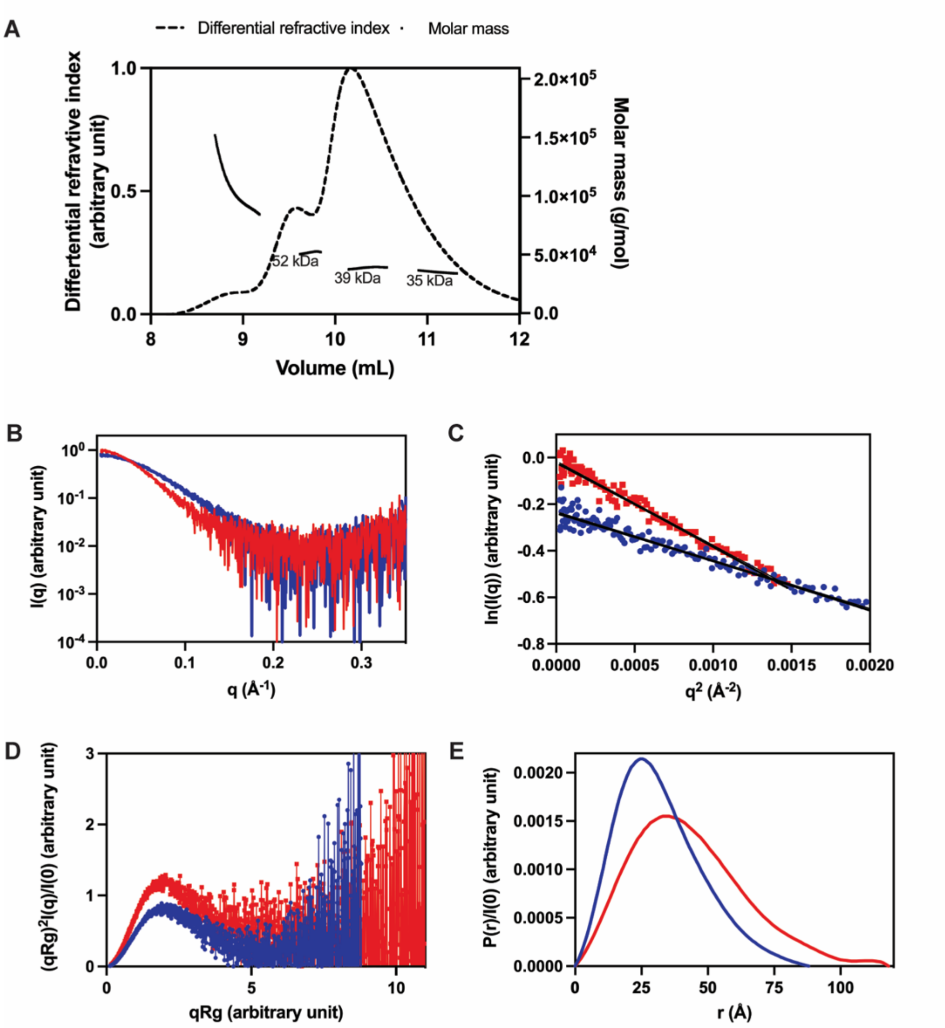

Fig. S9. SEC-MALS-SAXS analysis of SARS-CoV-2 nsp7-8 polyprotein. A) SEC-MALS chromatogram of nsp7-8 polyprotein. Dashed line represents the DRI (differential refractive index) signal, and the plain line represents the molecular weight of the corresponding peaks. B) SAXS scattering profiles with I(q) versus q as log-linear plots, C) Guinier fit, D) Dimensionless Kratky plots and E) Pair-wise distribution curve of monomeric (blue) and dimeric (red) nsp7-8 polyprotein.

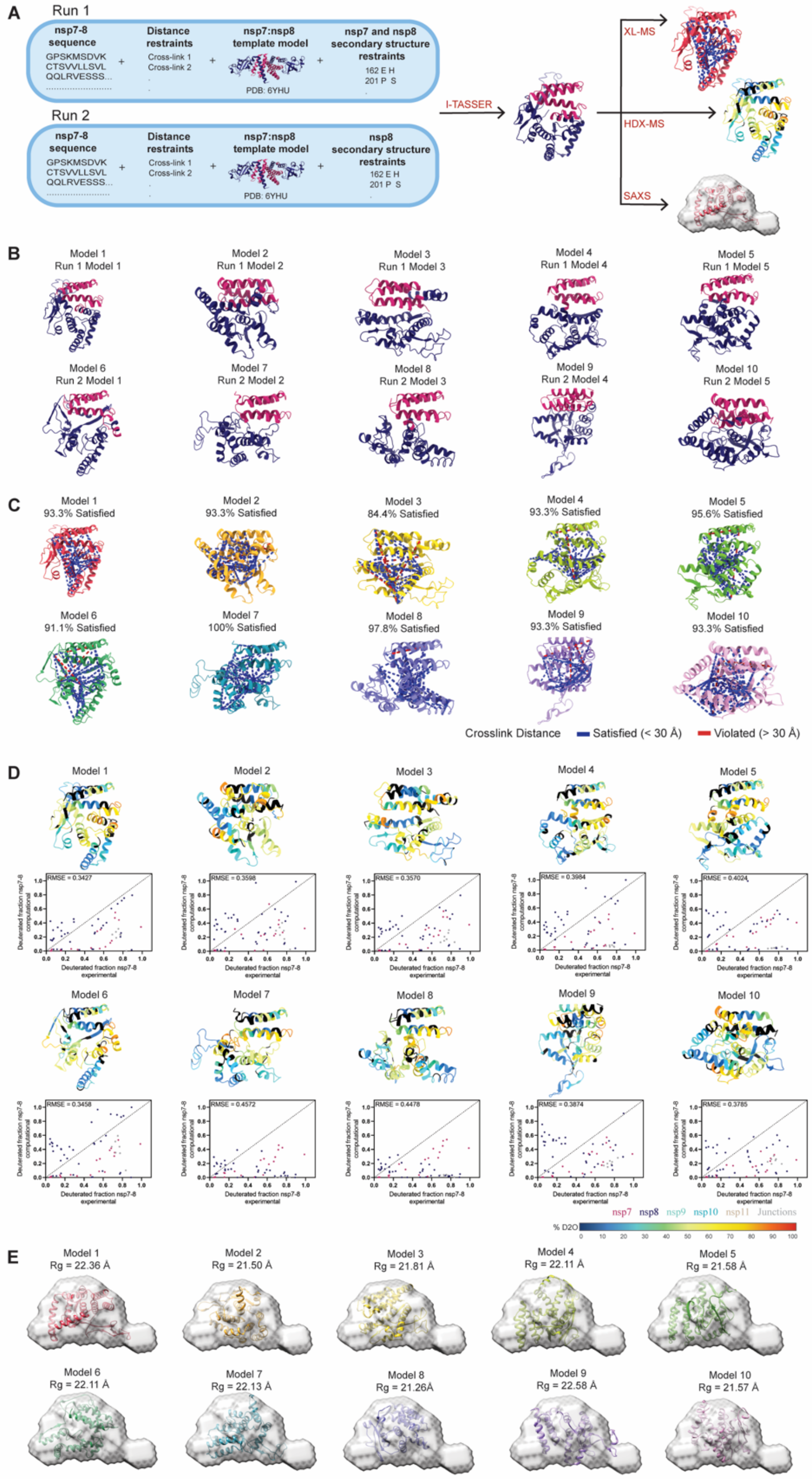

F

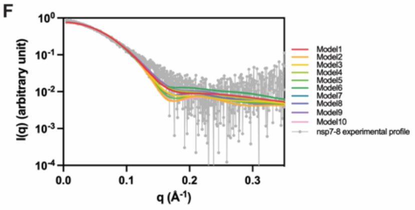

	χ^2
Model 1	2.16
Model 2	3.17
Model 3	2.67
Model 4	2.25
Model 5	2.94
Model 6	2.51
Model 7	2.17
Model 8	3.04
Model 9	1.91
Model 10	3.02

Fig. S10. Integrative structural modeling approach and assessment of SARS-CoV-2 nsp7-8 top ten models. (A) Scheme of integrative structural modeling workflow for nsp7-8 polyprotein. One model is shown to represent all ten generated. (B) Top ten nsp7-8 models with nsp7 colored in magenta and nsp8 in dark purple. (C) Mapping nsp7-8 intra-protein crosslinks onto all nsp7-8 models. Satisfied crosslinks equal to or less than 30 Å are shown in blue and violated crosslinks greater than 30 Å are shown in red. Percent of crosslinks satisfied is reported. (D) Top ten nsp7-8 models are colored based on percent deuterium value at 10 s incubation in deuterated buffer. Black indicates no sequence coverage in the HDX-MS profiling experiment. Plots of calculated 10 s deuterated fraction vs. experimental deuterated fraction shown under each corresponding model with agreement score reported as the RMSE value. (E) Fitting of all nsp7-8 models into the reconstructed SAXS envelope and Rg values are reported under the model. (F) Theoretical scattering profile of all nsp7-8 models fitted against the experimental profile with $\chi 2$ values of the fit reported.

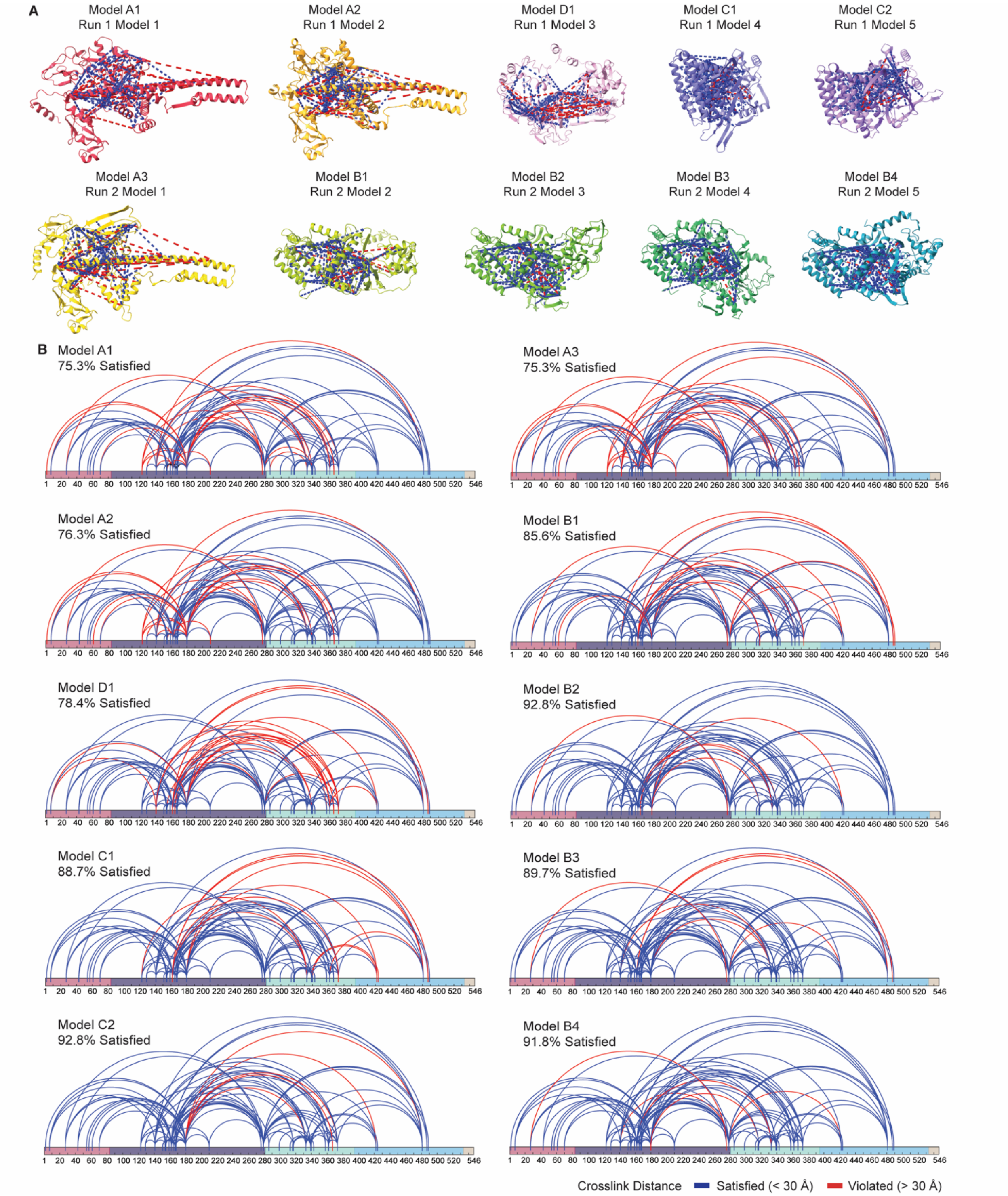
A
Model A1
Run 1 Model 1
Model A2
Run 1 Model 2
Model D1
Run 1 Model 3
Model C1
Run 1 Model 4
Model C2
Run 1 Model 5
Model A3
Run 2 Model 1
Model B1
Run 2 Model 2
Model B2
Run 2 Model 3
Model B3
Run 2 Model 4
Model B4
Run 2 Model 5
B
Model A1
75.3% Satisfied
Model A3
75.3% Satisfied
Model A2
76.3% Satisfied
Model B1
85.6% Satisfied
Model D1
78.4% Satisfied
Model B2
92.8% Satisfied
Model C1
88.7% Satisfied
Model B3
89.7% Satisfied
Model C2
92.8% Satisfied
Model B4
91.8% Satisfied
1 20 40 60 80 100 120 140 160 180 200 220 240 260 280 300 320 340 360 380 400 420 440 460 480 500 520 546
Crosslink Distance
Satisfied (< 30 Å)
Violated (> 30 Å)

Fig. S11. Assessment of SARS-CoV-2 nsp7-11 top ten models based on XL-MS data. (A) Top ten nsp7-11 models with all nsp7-11 intra-protein crosslinks mapped. Satisfied crosslinks equal to or less than 30 Å are shown in blue and violated crosslinks greater than 30 Å are shown in red. (B) Alternative view of all nsp7-11 intra-protein crosslinks mapped onto nsp7-11 sequence with nsp7 in magenta, nsp8 in purple, nsp9 in teal, nsp10 in cyan and nsp11 in tan. Percent of crosslinks satisfied is reported for each model.

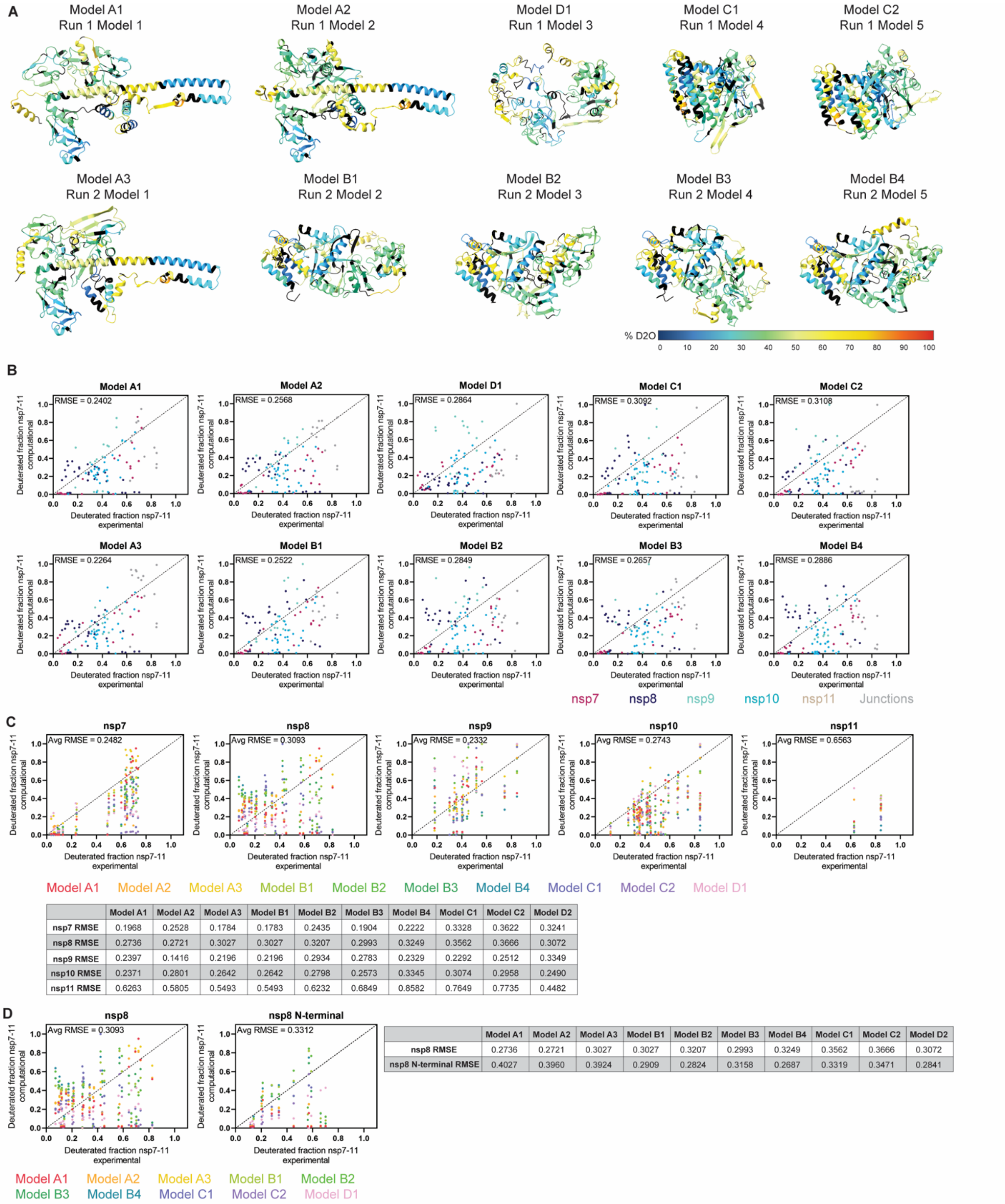

	Model A1	Model A2	Model A3	Model B1	Model B2	Model B3	Model B4	Model C1	Model C2	Model D2
nsp7 RMSE	0.1968	0.2528	0.1784	0.1783	0.2435	0.1904	0.2222	0.3328	0.3622	0.3241
nsp8 RMSE	0.2736	0.2721	0.3027	0.3027	0.3207	0.2993	0.3249	0.3562	0.3666	0.3072
nsp9 RMSE	0.2397	0.1416	0.2196	0.2196	0.2934	0.2783	0.2329	0.2292	0.2512	0.3349
nsp10 RMSE	0.2371	0.2801	0.2642	0.2642	0.2798	0.2573	0.3345	0.3074	0.2958	0.2490
nsp11 RMSE	0.6263	0.5805	0.5493	0.5493	0.6232	0.6849	0.8582	0.7649	0.7735	0.4482

	Model A1	Model A2	Model A3	Model B1	Model B2	Model B3	Model B4	Model C1	Model C2	Model D2
nsp8 RMSE	0.2736	0.2721	0.3027	0.3027	0.3207	0.2993	0.3249	0.3562	0.3666	0.3072
nsp8 N-terminal RMSE	0.4027	0.3960	0.3924	0.2909	0.2824	0.3158	0.2687	0.3319	0.3471	0.2841

Fig. S12. Assessment of SARS-CoV-2 nsp7-11 top ten models based on HDX-MS data. (A) Top ten nsp7-11 models colored based on percent deuterium value after 10 s incubation in deuterated buffer. Black indicates no sequence coverage in the HDX-MS experiment. (B) Plots of calculated 10 s deuterated fraction for each model versus experimental deuterated fraction with

agreement score reported as the RMSE value. Data points are colored based on peptide sequence with nsp7 in magenta, nsp8 in purple, nsp9 in teal, nsp10 in cyan, nsp11 in tan, and junctions in gray. (C) Plots of calculated 10 s deuterated fraction for each nsp versus experimental deuterated fraction. Data points are colored based on models. The RMSE value per each individual nsp for each model is reported in the table below. The average agreement scores of given individual nsp for all models is reported as the average RMSE value on plot. (D) Comparison of agreement of nsp8 and nsp8 N-terminal (residues 92-213) only to experimental HDX-MS data. Plots showing calculated 10 s deuterated fraction versus experimental deuterated fraction with data points are colored based on models. The RMSE value for each model is reported in the table below and the average agreement score for all models is reported as the average RMSE value on plot.

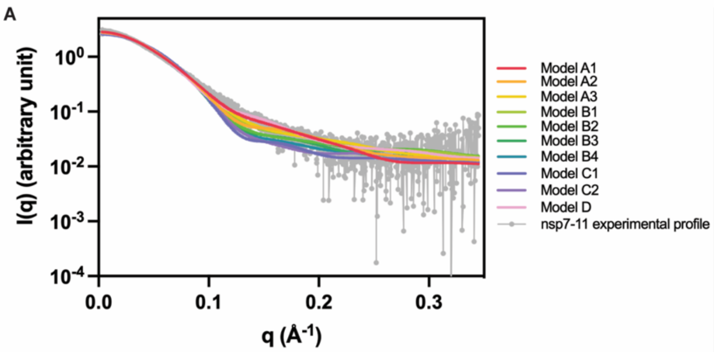

	χ^2
Model A1	6.42
Model A2	15.53
Model A3	7.89
Model B1	20.76
Model B2	29.12
Model B3	31.48
Model B4	34.55
Model C1	49.00
Model C2	40.91
Model D	18.31

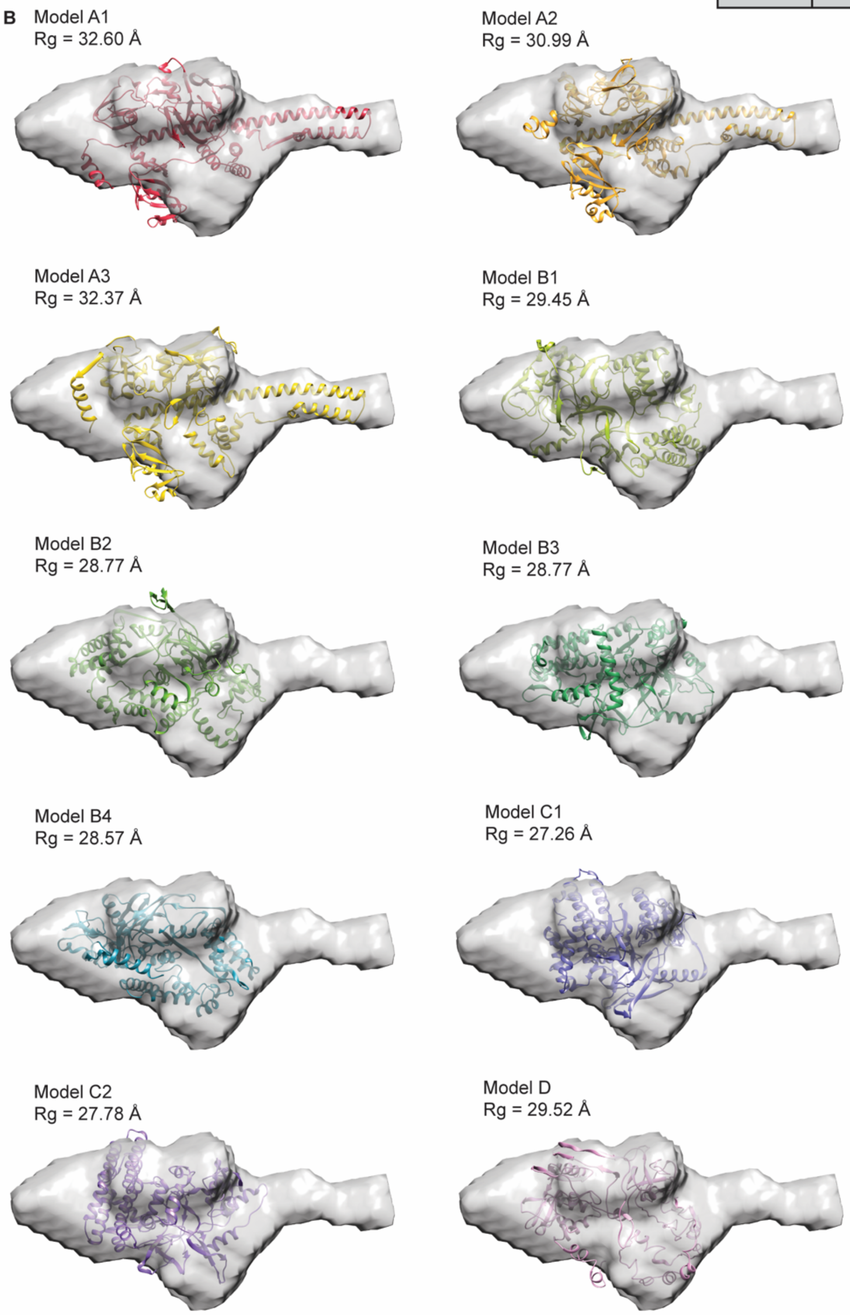

Fig. S13. Assessment of SARS-CoV-2 nsp7-11 top ten models based on SAXS. (A) Theoretical scattering profile of top 10 models fitted against the experimental profile with $\chi2$ values. (B) All models fitted in the reconstructed SAXS envelopes with Rg value reported for each model. The theoretical Rg value for the models and the $\chi2$ of the fit are calculated using *CRYSOL*.

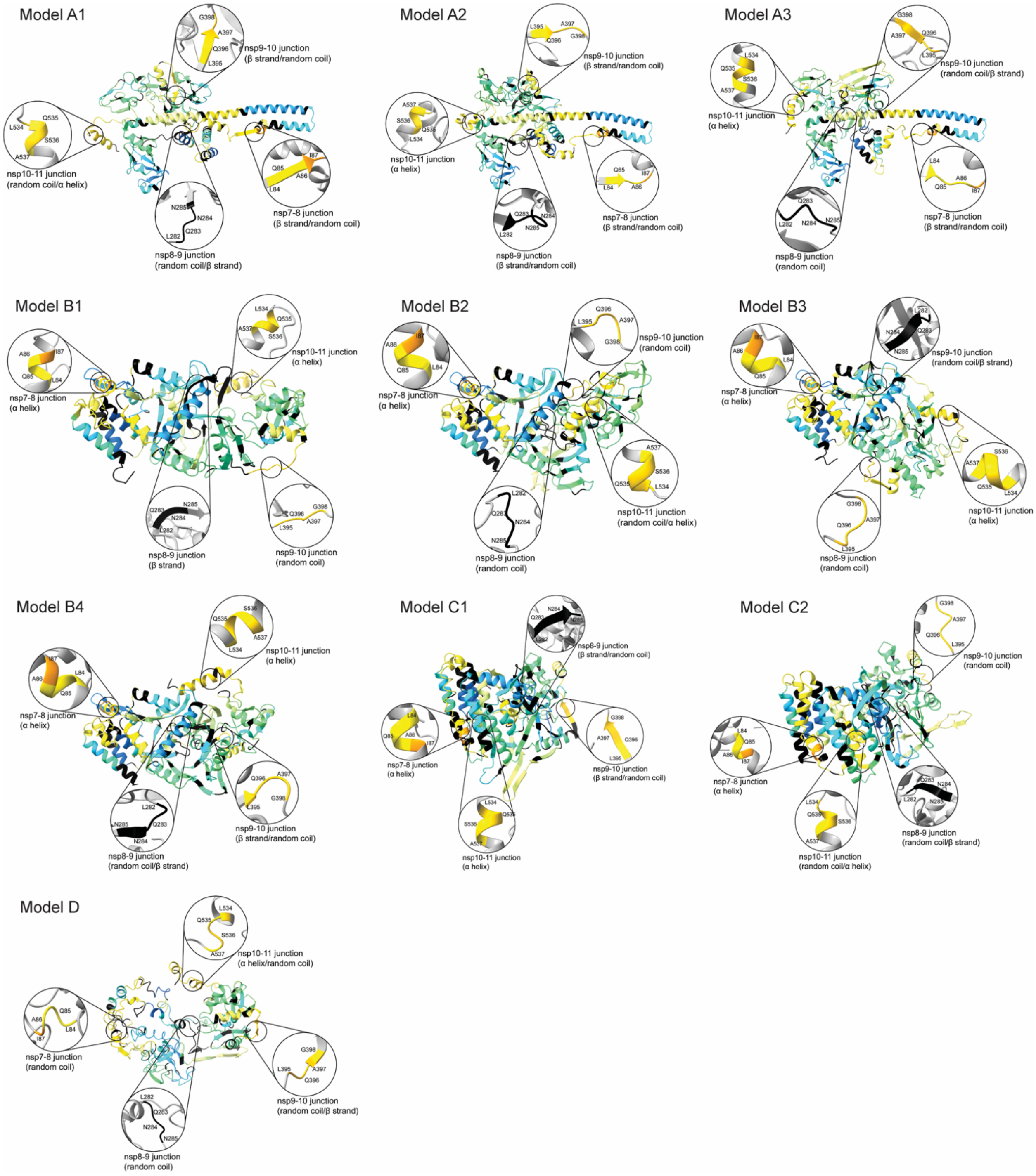

Fig. S14. Assessment of the junction sites of top ten models of SARS-CoV-2 nsp7-11. Analysis of the secondary structure elements of the junction sites in all nsp7-11 models. Models are overlaid with percent deuterium uptake values from **Fig. S12A**.

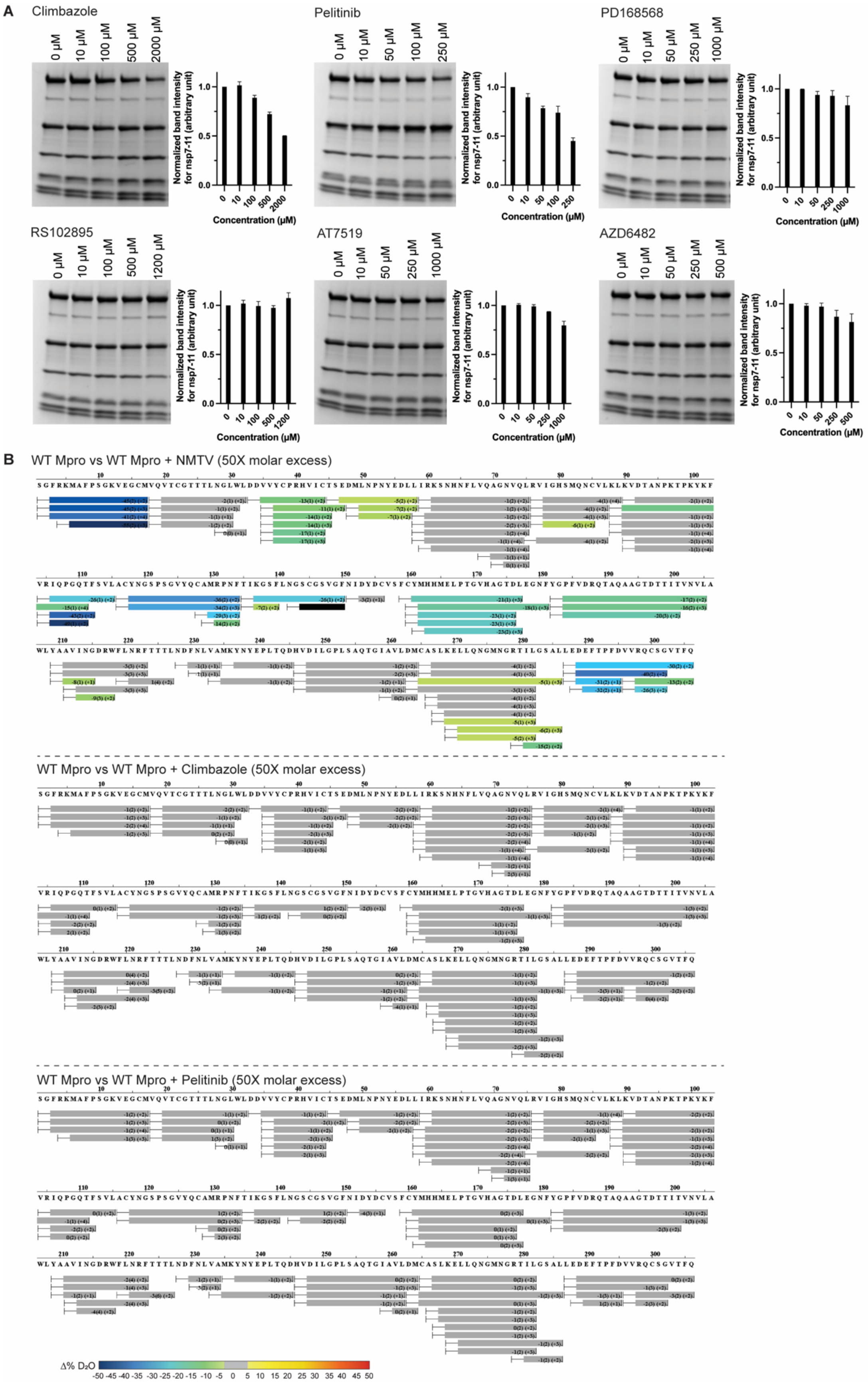
A
Climbazole
0 µM
10 µM
100 µM
500 µM
2000 µM
Normalized band intensity for nsp7-11 (arbitrary unit)
Concentration (µM)
Pelitinib
0 µM
10 µM
50 µM
100 µM
250 µM
PD168568
0 µM
10 µM
50 µM
250 µM
1000 µM
RS102895
0 µM
10 µM
100 µM
500 µM
1200 µM
AT7519
0 µM
10 µM
50 µM
250 µM
1000 µM
AZD6482
0 µM
10 µM
50 µM
250 µM
500 µM
B
WT Mpro vs WT Mpro + NMTV (50X molar excess)
WT Mpro vs WT Mpro + Climbazole (50X molar excess)
WT Mpro vs WT Mpro + Pelitinib (50X molar excess)
Δ% D₂O
-50 -45 -40 -35 -30 -25 -20 -15 -10 -5 0 5 10 15 20 25 30 35 40 45 50

Fig. S15. Analysis of Mpro crystallographic binders and NMTV effects on SARS-CoV-2 nsp7-11 polyprotein processing by WT Mpro. (A) SDS-PAGE gels of nsp7-11 polyprotein processing by WT Mpro in the presence of small molecule binders after 24 h. The band intensities were calculated for the nsp7-11 gel band using ImageJ and plotted against the concentration of the binders. The identity of the substrate, intermediate, and final cleavage product gel bands can be referenced to **Fig. S2**. (B) Sequence coverage of differential HDX-MS analysis of WT Mpro versus WT Mpro with binders at 12 h incubation in deuterated buffer only. Color scale represents changes in deuterium uptake with gray representing no significant change in deuterium uptake.

Table S1. HDX-MS experimental conditions and data analysis parameters from the guidelines of the IC-HDX-MS community.

**Data Set (Figure 1C, S5)**	**nsp7-11**				**nsp7-11 : WT Mpro**		
Reaction details	50 mM HEPES, 500 mM NaCl, 1 mM TCEP, pH 8.0, 4 °C						
	600 s	1800 s	3600 s	7200 s	14400 s	28800 s	86400 s
Deuterium labeling time (sec)	30						
HDX quench reaction details	5 M urea, 1% TFA, pH = 2.0, 4 °C						
Back-exchange	estimated from input recovery estimate of 0.7 and deuterated buffer concentration of 0.8						
# of peptides	119						
Sequence coverage	99%						
Average peptide length / Redundancy	13 / 3.314						
Replicates (biological or technical)	3 (technical)						
Repeatability (average STD)	1.478	1.811	1.683	1.460	1.984	2.038	2.163
Significant differences in HDX	> 5% D (unpaired t-tests at each time point, p-value < 0.01)						
**Data Set (Figure S6)**	**nsp7-10**			**nsp7-10 : WT Mpro**			
Reaction details	50 mM HEPES, 500 mM NaCl, 1 mM TCEP, pH 8.0, 4 °C						
	600 s	1800 s	3600 s	14400 s	86400 s		
Deuterium labeling time (sec)	30						
HDX quench reaction details	5 M urea, 1% TFA, pH = 2.0, 4 °C						
Back-exchange	estimated from input recovery estimate of 0.7 and deuterated buffer concentration of 0.8						
# of peptides	107						
Sequence coverage	99%						
Average peptide length / Redundancy	10 / 3.110						
Replicates (biological or technical)	3 (technical)						
Repeatability (average STD)	1.723	1.563	1.743	1.722	2.511		
Significant differences in HDX	> 5% D (unpaired t-tests at each time point, p-value < 0.01)						
**Data Set (Figure 2A, S7A)**	**nsp7-11**			**nsp7-11 : C145A Mpro**			
Reaction details	50 mM HEPES, 500 mM NaCl, 1 mM TCEP, pH 8.0, 4 °C						
	30 min						
Deuterium labeling time (sec)	10, 30, 60, 900, 3600						
HDX quench reaction details	5 M urea, 1% TFA, pH = 2.0, 4 °C						
Back-exchange	estimated from input recovery estimate of 0.7 and deuterated buffer concentration of 0.8						
# of peptides	133						
Sequence coverage	93%						
Average peptide length / Redundancy	4 / 3.604						

Replicates (biological or technical)	3 (technical)	
Repeatability	4.178 (average STD)	
Significant differences in HDX	> 5% D (unpaired t-tests at each time point, p-value < 0.01)	
**Data Set (Figure 2B, 2F, S7B)**	**nsp7-10**	**nsp7-10 : C145A Mpro**
Reaction details	50 mM HEPES, 500 mM NaCl, 1 mM TCEP, pH 8.0, 4 °C	
	30 min	
Deuterium labeling time (sec)	10, 30, 60, 900, 3600	
HDX quench reaction details	5 M urea, 1% TFA, pH = 2.0, 4 °C	
Back-exchange	estimated from input recovery estimate of 0.7 and deuterated buffer concentration of 0.8	
# of peptides	97	
Sequence coverage	90%	
Average peptide length / Redundancy	9 / 2.842	
Replicates (biological or technical)	3 (technical)	
Repeatability (average STD)	3.454	
Significant differences in HDX	> 5% D (unpaired t-tests at each time point, p-value < 0.01)	
**Data Set (Figure 2C-D, 2E-F, 6A, S7C-D)**	**C145A Mpro**	**C145A Mpro : nsp7-11**
Reaction details	50 mM HEPES, 500 mM NaCl, 1 mM TCEP, pH 8.0, 4 °C	
	30 min	
Deuterium labeling time (sec)	10, 30, 60, 900, 3600, 43200	
HDX quench reaction details	5 M urea, 1% TFA, pH = 2.0, 4 °C	
Back-exchange	estimated from input recovery estimate of 0.7 and deuterated buffer concentration of 0.8	
# of peptides	87	
Sequence coverage	98%	
Average peptide length / Redundancy	8 / 4.073	
Replicates (biological or technical)	3 (technical)	
Repeatability (average STD)	3.542	
Significant differences in HDX	> 5% D (unpaired t-tests at each time point, p-value < 0.01)	
**Data Set (Figure 6E, S15Bi)**	**WT Mpro**	**WT Mpro + NMTV**
Reaction details	50 mM HEPES, 500 mM NaCl, 1 mM TCEP, pH 8.0, 4 °C	
	30 min	
Deuterium labeling time (sec)	43200	
HDX quench reaction details	5 M urea, 1% TFA, pH = 2.0, 4 °C	
Back-exchange	estimated from input recovery estimate of 0.7 and deuterated buffer concentration of 0.8	
# of peptides	90	
Sequence coverage	98%	

Average peptide length / Redundancy	8 / 4.163	
Replicates (biological or technical)	3 (technical)	
Repeatability (average STD)	1.594	
Significant differences in HDX	> 5 %D (unpaired t-tests at each time point, p-value < 0.01)	
**Data Set (Figure S15Bii)**	**WT Mpro**	**WT Mpro + Climbazole**
Reaction details	50 mM HEPES, 500 mM NaCl, 1 mM TCEP, pH 8.0, 4 °C	
	30 min	
Deuterium labeling time (sec)	43200	
HDX quench reaction details	5 M urea, 1% TFA, pH = 2.0, 4 °C	
Back-exchange	estimated from input recovery estimate of 0.7 and deuterated buffer concentration of 0.8	
# of peptides	90	
Sequence coverage	98%	
Average peptide length / Redundancy	8 / 4.163	
Replicates (biological or technical)	3 (technical)	
Repeatability (average STD)	1.808	
Significant differences in HDX	> 5% D (unpaired t-tests at each time point, p-value < 0.01)	
**Data Set (Figure S15Bii)**	**WT Mpro**	**WT Mpro + Pelitinib**
Reaction details	50 mM HEPES, 500 mM NaCl, 1 mM TCEP, pH 8.0, 4 °C	
	30 min	
Deuterium labeling time (sec)	43200	
HDX quench reaction details	5 M urea, 1% TFA, pH = 2.0, 4 °C	
Back-exchange	estimated from input recovery estimate of 0.7 and deuterated buffer concentration of 0.8	
# of peptides	90	
Sequence coverage	98%	
Average peptide length / Redundancy	8 / 4.163	
Replicates (biological or technical)	3 (technical)	
Repeatability (average STD)	2.001	
Significant differences in HDX	> 5% D (unpaired t-tests at each time point, p-value < 0.01)	

Table S2. SEC-MALS-SAXS table for SARS-CoV-2 nsp7-11 and nsp7-8 polyprotein samples.

**A) SAS data-collection parameters**				
Instrument	BioCAT facility at the Advanced Photon Source beamline 18ID with Pilatus3 X 1M (Dectris) detector			
Wavelength (Å)	1.033			
Beam size (μm^2)	150 (h) x 25 (v) focused at the detector			
Camera length (m)	3.69 m for nsp7-11 sample and 3.631 m for the nsp7-8 sample			
q range ($Å^{-1}$)	0.003 to 0.35			
Absolute scaling method	Glassy Carbon, NIST SRM 3600			
Basis for normalization to constant counts	To transmitted intensity by beam-stop counter			
Method for monitoring radiation damage	Automated frame-by-frame comparison of relevant regions using CORMAP (*88*) implemented in BioXTAS RAW			
Exposure time, number of exposures	0.5 s exposure time with a 1 s total exposure period (0.5 s on, 0.5 s off) of entire SEC elution			
Sample configuration	SEC-MALS-SAXS. Size separation used a Superdex 200 Increase 10/300 GL column and a 1260 Infinity II HPLC (Agilent Technologies). UV data was measured in the Agilent, and MALS-DLS-RI data by DAWN HELEOS-II (17 MALS + 1 DLS channels) and Optilab T-rEX (RI) instruments (Wyatt Technology). SAXS data were measured in a sheath-flow cell (*80*), effective path length 0.542 mm			
Sample temperature (ºC)	23			
**B) Software employed for SAS data reduction, analysis and interpretation**				
SAXS data reduction	Radial averaging; frame comparison, averaging, and subtraction done using BioXTAS RAW 2.0.3 (*81*)			
Basic analysis: Guinier, M.W., P(r)	Guinier fit and M.W. using BioXTAS RAW, P(r) function using GNOM (*89*). RAW uses MoW and Vc M.W. methods (*90, 91*)			
Shape/bead modelling	*DAMMIF* (*51*) and *DAMMIN* (*52*) via *ATSAS*			
MALS-DLS-RI analysis	Astra 7 (Wyatt)			
**C) Structural parameters**				
	**nsp7-11**		**nsp-8**	
	**monomer**	**dimer**	**monomer**	**dimer**
**Guinier analysis**				
I(0)	2.9 +/- 4.18e-3	0.53 +/- 2.73e-3	0.79 +/- 2.61e-3	0.99 +/- 3.21e-3

R_g (Å)	34.91 +/- 0.15	45.69 +/- 0.39	24.96 +/- 0.16	33.02 +/- 0.2
q_{min} ($Å^{-1}$)	0.00467	0.00297	0.00475	0.00475
qR_gmax	0.92	1.29	1.22	1.24
Correlation coefficient, R^2	0.99	0.75	0.97	0.97
M.W. (Bayes) [kDa]	62.4	91.2	33.1	62.4
**P(r) analysis**				
I(0)	2.9 +/- 4.28e-3	0.53 +/- 3.59e-3	0.79 +/- 2.47e-3	0.99 +/- 3.58e-3
R_g (Å)	35.89 +/- 0.15	48.16 +/- 0.61	25.24 +/- 0.13	33.69 +/- 0.24
d_{max} (Å)	156.0	191.0	88.0	118.0
q range ($Å^{-1}$)	0.005- 0.229	0.003- 0.175	0.005- 0.299	0.0047- 0.2424
χ^2 (total estimate from GNOM)	2.277 (0.719)	1.822 (0.771)	1.085 (0.829)	1.785 (0.793)
M.W. (Vp) [kDa]	72.7	130.8	34.4	70.9
Porod volume ($Å^3$)	8.75e+4	1.58e+5	4.15e+4	8.54e+4

**D) Shape model-fitting results**		
	**nsp7-11 (monomer)**	**nsp7-8 (monomer)**
***DAMMIF* (default parameters, 20 calculations)**		
q range for fitting ($Å^{-1}$)	0.005- 0.229	0.005- 0.299
Symmetry, anisotropy assumptions	P1, none	P1, none
NSD (standard deviation), No. of clusters	0.951 (0.057), 9	1.087 (0.180), 7
χ^2 range	2.303-2.318	1.092-1.093
Resolution (from SASRES) (Å)	42 +/- 3 Å	27 +/- 2 Å
M.W. estimate (kDa)	80.4	39.8
DAMMIN (default parameters)		
q range for fitting ($Å^{-1}$)	0.005- 0.229	0.005- 0.299
Symmetry, anisotropy assumptions	P1, none	P1, none
χ^2	2.278	1.087

Table S3. Summary of agreement of top ten nsp7-11 models with experimental data from HDX-MS, XL-MS, SAXS, and junction accessible surface area.

		Model A1	Model A2	Model A3	Model B1	Model B2	Model B3	Model B4	Model C1	Model C2	Model D1
**RMSE from HDXer**		0.2402	0.2568	0.2264	0.2522	0.2849	0.2657	0.2886	0.3092	0.3108	0.2864
**% XL satisfied (≤ 30 Å)**		75.3	76.3	75.3	85.6	92.8	89.7	91.8	88.7	92.8	78.4
**Rg (Å)**		32.60	30.99	32.37	29.45	28.77	28.77	28.57	27.26	27.78	29.52
χ^2		6.42	15.53	7.89	20.76	29.12	31.48	34.55	49.0	40.91	18.31
**Junction Accessible Surface Area ($Å^2$)**	**nsp7-8 site**	414.7	425.9	393.4	155.7	161.3	160.0	155.5	142.5	182.3	141.6
	**nsp8-9 site**	280.6	276.3	201.9	353.2	138.7	251.4	209.8	97.3	129.9	208.6
	**nsp9-10 site**	219.3	324.7	308.2	306.5	336.0	340.1	232.9	440.1	357.4	420.8
	**nsp10-11 site**	248.2	254.1	270.6	292.1	396.1	273.4	223.2	247.0	310.1	261.5

Table S4. SARS-CoV-2 Mpro crystallographic binders interacting in various regions of Mpro showing protection from solvent exchange in differential HDX-MS of C145A Mpro versus C145A Mpro with nsp7-11 at 12 h incubation in deuterated buffer only.

PDB code	PDB chains	Ligand PDB code	Region of Mpro	Fragment or Small molecule (name)	Antiviral activity	Pubchem CID	PMID
5RE5	A	T0J_A_404	Back of the catalytic domain	Fragment	ND	769265	33028810
5RE6	A	O0S_A_404		Fragment	ND	1487531	33028810
5RFB	A	K3S_A_404		Fragment	ND	62755740	33028810
5RFC	A	K1Y_A_404		Fragment	ND	811874	33028810
5RGG	A	NZD_A_404		Fragment	ND	762797	33028810
5RH4	A	UHG_A_1001		Fragment	ND	6997572	33028810
6YVF	**A**	**A82_A_401**		**AZD6482**	**NA**	**44137675**	**33811162**
5REF	A	6SU_A_404	Near dimerization domain	Fragment	ND	2806372	33028810
**7AGA**	**A**	**LZE_A_401**		**AT7519**	**EC_{50} = 25 µM**	**11338033**	**33811162**
5REG	A	LWA_A_404	Surface pocket	Fragment	ND	1086839	33028810
**7AOL**	**A**	**RQH_A_403**		**climbazole**	**NA**	**25271637**	**33811162**
5RFA	A	JGY_A_403	Dimerization domain	Fragment	ND	1224835	33028810
**7ABU**	**A**	**R6Q_A_401**		**RS102895**	**EC_{50} = 19.8 µM**	**10000456**	**33811162**
**7AMJ**	**A**	**RMZ_A_409**		**PD 168568**	**NA**	**9798466**	**33811162**
**7AXM**	**A**	**93J_A_502**		**pelitinib**	**EC_{50} = 1.25 µM**	**6445562**	**33811162**
7BFB	B	9JT_B_1005		Ebselen	EC_{50} = 4.67 µM*	126410	32272481

ND = not determined; NA = no activity; ligands assayed in bold; *this small molecule binds in two more sites in Mpro; ligands in bold were tested in limited proteolysis assay and colored according to **Fig. 6A**.

REFERENCES AND NOTES

1. N. Zhu, D. Zhang, W. Wang, X. Li, B. Yang, J. Song, X. Zhao, B. Huang, W. Shi, R. Lu, P. Niu, F. Zhan, X. Ma, D. Wang, W. Xu, G. Wu, G. F. Gao, W. Tan; China Novel Coronavirus Investigating and Research Team, A novel coronavirus from patients with pneumonia in China, 2019. *N. Engl. J. Med.* **382**, 727–733 (2020).

2. D. E. Gordon, G. M. Jang, M. Bouhaddou, J. Xu, K. Obernier, K. M. White, M. J. O'Meara, V. V. Rezelj, J. Z. Guo, D. L. Swaney, T. A. Tummino, R. Hüttenhain, R. M. Kaake, A. L. Richards, B. Tutuncuoglu, H. Foussard, J. Batra, K. Haas, M. Modak, M. Kim, P. Haas, B. J. Polacco, H. Braberg, J. M. Fabius, M. Eckhardt, M. Soucheray, M. J. Bennett, M. Cakir, M. J. McGregor, Q. Li, B. Meyer, F. Roesch, T. Vallet, A. M. Kain, L. Miorin, E. Moreno, Z. Z. C. Naing, Y. Zhou, S. Peng, Y. Shi, Z. Zhang, W. Shen, I. T. Kirby, J. E. Melnyk, J. S. Chorba, K. Lou, S. A. Dai, I. Barrio-Hernandez, D. Memon, C. Hernandez-Armenta, J. Lyu, C. J. P. Mathy, T. Perica, K. B. Pilla, S. J. Ganesan, D. J. Saltzberg, R. Rakesh, X. Liu, S. B. Rosenthal, L. Calviello, S. Venkataramanan, J. Liboy-Lugo, Y. Lin, X. P. Huang, Y. Liu, S. A. Wankowicz, M. Bohn, M. Safari, F. S. Ugur, C. Koh, N. S. Savar, Q. D. Tran, D. Shengjuler, S. J. Fletcher, M. C. O'Neal, Y. Cai, J. C. J. Chang, D. J. Broadhurst, S. Klippsten, P. P. Sharp, N. A. Wenzell, D. Kuzuoglu-Ozturk, H. Y. Wang, R. Trenker, J. M. Young, D. A. Cavero, J. Hiatt, T. L. Roth, U. Rathore, A. Subramanian, J. Noack, M. Hubert, R. M. Stroud, A. D. Frankel, O. S. Rosenberg, K. A. Verba, D. A. Agard, M. Ott, M. Emerman, N. Jura, M. von Zastrow, E. Verdin, A. Ashworth, O. Schwartz, C. d'Enfert, S. Mukherjee, M. Jacobson, H. S. Malik, D. G. Fujimori, T. Ideker, C. S. Craik, S. N. Floor, J. S. Fraser, J. D. Gross, A. Sali, B. L. Roth, D. Ruggero, J. Taunton, T. Kortemme, P. Beltrao, M. Vignuzzi, A. Garcia-Sastre, K. M. Shokat, B. K. Shoichet, N. J. Krogan, A SARS-CoV-2 protein interaction map reveals targets for drug repurposing. *Nature* **583**, 459–468 (2020).

3. B. Meyer, J. Chiaravalli, S. Gellenoncourt, P. Brownridge, D. P. Bryne, L. A. Daly, A. Grauslys, M. Walter, F. Agou, L. A. Chakrabarti, C. S. Craik, C. E. Eyers, P. A. Eyers, Y. Gambin, A. R. Jones, E. Sierecki, E. Verdin, M. Vignuzzi, E. Emmott, Characterising proteolysis during SARS-CoV-2 infection identifies viral cleavage sites and cellular targets with therapeutic potential. *Nat. Commun.* **12**, 5553 (2021).

4. E. Emmott, A. de Rougemont, M. Hosmillo, J. Lu, T. Fitzmaurice, J. Haas, I. Goodfellow, Polyprotein processing and intermolecular interactions within the viral replication complex spatially and temporally control norovirus protease activity. *J. Biol. Chem.* **294**, 4259–4271 (2019).

5. G. Shin, S. A. Yost, M. T. Miller, E. J. Elrod, A. Grakoui, J. Marcotrigiano, Structural and functional insights into alphavirus polyprotein processing and pathogenesis. *Proc. Natl. Acad. Sci. U.S.A.* **109**, 16534–16539 (2012).

6. S. A. Yost, J. Marcotrigiano, Viral precursor polyproteins: Keys of regulation from replication to maturation. *Curr. Opin. Virol.* **3**, 137–142 (2013).

7. A. G. Bost, R. H. Carnahan, X. T. Lu, M. R. Denison, Four proteins processed from the replicase gene polyprotein of mouse hepatitis virus colocalize in the cell periphery and adjacent to sites of virion assembly. *J. Virol.* **74**, 3379–3387 (2000).

8. D. J. Deming, R. L. Graham, M. R. Denison, R. S. Baric, Processing of open reading frame 1a replicase proteins nsp7 to nsp10 in murine hepatitis virus strain A59 replication. *J. Virol.* **81**, 10280–10291 (2007).

9. J. Ziebuhr, S. G. Siddell, Processing of the human coronavirus 229E replicase polyproteins by the virus-encoded 3C-like proteinase: Identification of proteolytic products and cleavage sites common to pp1a and pp1ab. *J. Virol.* **73**, 177–185 (1999).

10. W. Cui, S. Cui, C. Chen, X. Chen, Z. Wang, H. Yang, L. Zhang, The crystal structure of main protease from mouse hepatitis virus A59 in complex with an inhibitor. *Biochem. Biophys. Res. Commun.* **511**, 794–799 (2019).

11. R. W. Körner, M. Majjouti, M. A. A. Alcazar, E. Mahabir, Of mice and men: The coronavirus MHV and mouse models as a translational approach to understand SARS-CoV-2. *Viruses* **12**, 880 (2020).

12. T. P. Sheahan, A. C. Sims, S. T. Zhou, R. L. Graham, A. J. Pruijssers, M. L. Agostini, S. R. Leist, A. Schafer, K. H. Dinnon, L. J. Stevens, J. D. Chappell, X. T. Lu, T. M. Hughes, A. S.

George, C. S. Hill, S. A. Montgomery, A. J. Brown, G. R. Bluemling, M. G. Natchus, M. Saindane, A. A. Kolykhalov, G. Painter, J. Harcourt, A. Tamin, N. J. Thornburg, R. Swanstrom, M. R. Denison, R. S. Baric, An orally bioavailable broad-spectrum antiviral inhibits SARS-CoV-2 in human airway epithelial cell cultures and multiple coronaviruses in mice. *Sci. Transl. Med.* **12**, eabb5883 (2020).

13. A. Kanjanahaluethai, S. C. Baker, Identification of mouse hepatitis virus papain-like proteinase 2 activity. *J. Virol.* **74**, 7911–7921 (2000).

14. J. J. Schiller, A. Kanjanahaluethai, S. C. Baker, Processing of the coronavirus MHV-JHM polymerase polyprotein: Identification of precursors and proteolytic products spanning 400 kilodaltons of ORF1a. *Virology* **242**, 288–302 (1998).

15. S. G. Sawicki, D. L. Sawicki, D. Younker, Y. Meyer, V. Thiel, H. Stokes, S. G. Siddell, Functional and genetic analysis of coronavirus replicase-transcriptase proteins. *PLOS Pathog.* **1**, e39 (2005).

16. E. F. Donaldson, R. L. Graham, A. C. Sims, M. R. Denison, R. S. Baric, Analysis of murine hepatitis virus strain A59 temperature-sensitive mutant TS-LA6 suggests that nsp10 plays a critical role in polyprotein processing. *J. Virol.* **81**, 7086–7098 (2007).

17. R. Gosert, A. Kanjanahaluethai, D. Egger, K. Bienz, S. C. Baker, RNA replication of mouse hepatitis virus takes place at double-membrane vesicles. *J. Virol.* **76**, 3697–3708 (2002).

18. K. Knoops, M. Kikkert, S. H. E. van den Worm, J. C. Zevenhoven-Dobbe, Y. van der Meer, A. J. Koster, A. M. Mommaas, E. J. Snijder, SARS-coronavirus replication is supported by a reticulovesicular network of modified endoplasmic reticulum. *PLOS Biol.* **6**, 1957–1974 (2008).

19. D. Oudshoorn, K. Rijs, R. W. A. L. Limpens, K. Groen, A. J. Koster, E. J. Snijder, M. Kikkert, M. Barcena, Expression and cleavage of middle east respiratory syndrome coronavirus nsp3-4 polyprotein induce the formation of double-membrane vesicles that mimic those associated with coronaviral RNA replication. *mBio* **8**, e01658-17 (2017).

20. G. Wolff, R. W. A. L. Limpens, J. C. Zevenhoven-Dobbe, U. Laugks, S. Zheng, A. W. M. de Jong, R. I. Koning, D. A. Agard, K. Grunewald, A. J. Koster, E. J. Snijder, M. Barcena, A molecular pore spans the double membrane of the coronavirus replication organelle. *Science* **369**, 1395–1398 (2020).

21. B. Krichel, G. Bylapudi, C. Schmidt, C. Blanchet, R. Schubert, L. Brings, M. Koehler, R. Zenobi, D. Svergun, K. Lorenzen, R. Madhugiri, J. Ziebuhr, C. Uetrecht, Hallmarks of *Alpha*- and *Betacoronavirus* non-structural protein 7+8 complexes. *Sci. Adv.* **7**, eabf1004 (2021).

22. B. Krichel, S. Falke, R. Hilgenfeld, L. Redecke, C. Uetrecht, Processing of the SARS-CoV pp1a/ab nsp7-10 region. *Biochem. J.* **477**, 1009–1019 (2020).

23. S. Gildenhuys, Expanding our understanding of the role polyprotein conformation plays in the coronavirus life cycle. *Biochem. J.* **477**, 1479–1482 (2020).

24. G. D. Noske, A. M. Nakamura, V. O. Gawriljuk, R. S. Fernandes, G. M. A. Lima, H. V. D. Rosa, H. D. Pereira, A. C. M. Zeri, A. F. Z. Nascimento, M. C. L. C. Freire, D. Fearon, A. Douangamath, F. von Delft, G. Oliva, A. S. Godoy, A crystallographic snapshot of SARS-CoV-2 main protease maturation process. *J. Mol. Biol.* **433**, 167118 (2021).

25. D. W. Kneller, G. Phillips, H. M. O'Neill, R. Jedrzejczak, L. Stols, P. Langan, A. Joachimiak, L. Coates, A. Kovalevsky, Structural plasticity of SARS-CoV-2 3CL M^{pro} active site cavity revealed by room temperature x-ray crystallography. *Nat. Commun.* **11**, 3202 (2020).

26. D. W. Kneller, G. Phillips, H. M. O'Neill, K. Tan, A. Joachimiak, L. Coates, A. Kovalevsky, Room-temperature x-ray crystallography reveals the oxidation and reactivity of cysteine residues in SARS-CoV-2 3CL M^{pro}: Insights into enzyme mechanism and drug design. *IUCrJ* **7**, 1028–1035 (2020).

27. Z. M. Jin, X. Y. Du, Y. C. Xu, Y. Q. Deng, M. Q. Liu, Y. Zhao, B. Zhang, X. F. Li, L. K. Zhang, C. Peng, Y. K. Duan, J. Yu, L. Wang, K. L. Yang, F. J. Liu, R. D. Jiang, X. L. Yang, T. You, X. C. Liu, X. N. Yang, F. Bai, H. Liu, X. Liu, L. W. Guddat, W. Q. Xu, G. F. Xiao,

C. F. Qin, Z. L. Shi, H. L. Jiang, Z. H. Rao, H. T. Yang, Structure of Mpro from SARS-CoV-2 and discovery of its inhibitors. *Nature* **582**, 289–293 (2020).

28. L. L. Zhang, D. Z. Lin, X. Y. Y. Sun, U. Curth, C. Drosten, L. Sauerhering, S. Becker, K. Rox, R. Hilgenfeld, Crystal structure of SARS-CoV-2 main protease provides a basis for design of improved α-ketoamide inhibitors. *Science* **368**, 409–412 (2020).

29. D. R. Owen, C. M. N. Allerton, A. S. Anderson, L. Aschenbrenner, M. Avery, S. Berritt, B. Boras, R. D. Cardin, A. Carlo, K. J. Coffman, A. Dantonio, L. Di, H. Eng, R. Ferre, K. S. Gajiwala, S. A. Gibson, S. E. Greasley, B. L. Hurst, E. P. Kadar, A. S. Kalgutkar, J. C. Lee, J. Lee, W. Liu, S. W. Mason, S. Noell, J. J. Novak, R. S. Obach, K. Ogilvie, N. C. Patel, M. Pettersson, D. K. Rai, M. R. Reese, M. F. Sammons, J. G. Sathish, R. S. P. Singh, C. M. Steppan, A. Stewart, J. B. Tuttle, L. Updyke, P. R. Verhoest, L. Q. Wei, Q. Y. Yang, Y. A. Zhu, An oral SARS-CoV-2 M-pro inhibitor clinical candidate for the treatment of COVID-19. *Science* **374**, 1586–1593 (2021).

30. A. Douangamath, D. Fearon, P. Gehrtz, T. Krojer, P. Lukacik, C. D. Owen, E. Resnick, C. Strain-Damerell, A. Aimon, P. Abranyi-Balogh, J. Brandao-Neto, A. Carbery, G. Davison, A. Dias, T. D. Downes, L. Dunnett, M. Fairhead, J. D. Firth, S. P. Jones, A. Keeley, G. M. Keseru, H. F. Klein, M. P. Martin, M. E. M. Noble, P. O'Brien, A. Powell, R. N. Reddi, R. Skyner, M. Snee, M. J. Waring, C. Wild, N. London, F. von Delft, M. A. Walsh, Crystallographic and electrophilic fragment screening of the SARS-CoV-2 main protease. *Nat. Commun.* **11**, 5047 (2020).

31. N. Drayman, J. K. DeMarco, K. A. Jones, S. A. Azizi, H. M. Froggatt, K. Tan, N. I. Maltseva, S. Chen, V. Nicolaescu, S. Dvorkin, K. Furlong, R. S. Kathayat, M. R. Firpo, V. Mastrodomenico, E. A. Bruce, M. M. Schmidt, R. Jedrzejczak, M. A. Munoz-Alia, B. Schuster, V. Nair, K. Y. Han, A. O'Brien, A. Tomatsidou, B. Meyer, M. Vignuzzi, D. Missiakas, J. W. Botten, C. B. Brooke, H. Lee, S. C. Baker, B. C. Mounce, N. S. Heaton, W. E. Severson, K. E. Palmer, B. C. Dickinson, A. Joachimiak, G. Randall, S. Tay, Masitinib is a broad coronavirus 3CL inhibitor that blocks replication of SARS-CoV-2. *Science* **373**, 931–936 (2021).

32. S. Gunther, P. Y. A. Reinke, Y. Fernandez-Garcia, J. Lieske, T. J. Lane, H. M. Ginn, F. H. M. Koua, C. Ehrt, W. Ewert, D. Oberthuer, O. Yefanov, S. Meier, K. Lorenzen, B. Krichel, J. D. Kopicki, L. Gelisio, W. Brehm, I. Dunkel, B. Seychell, H. Gieseler, B. Norton-Baker, B. Escudero-Perez, M. Domaracky, S. Saouane, A. Tolstikova, T. A. White, A. Hanle, M. Groessler, H. Fleckenstein, F. Trost, M. Galchenkova, Y. Gevorkov, C. F. Li, S. Awel, A. Peck, M. Barthelmess, F. Schlunzen, P. L. Xavier, N. Werner, H. Andaleeb, N. Ullah, S. Falke, V. Srinivasan, B. A. Franca, M. Schwinzer, H. Brognaro, C. Rogers, D. Melo, J. J. Zaitseva-Doyle, J. Knoska, G. E. Pena-Murillo, A. R. Mashhour, V. Hennicke, P. Fischer, J. Hakanpaa, J. Meyer, P. Gribbon, B. Ellinger, M. Kuzikov, M. Wolf, A. R. Beccari, G. Bourenkov, D. von Stetten, G. Pompidor, I. Bento, S. Panneerselvam, I. Karpics, T. R. Schneider, M. M. Garcia-Alai, S. Niebling, C. Gunther, C. Schmidt, R. Schubert, H. J. Han, J. Boger, D. C. F. Monteiro, L. L. Zhang, X. Y. Y. Sun, J. Pletzer-Zelgert, J. Wollenhaupt, C. G. Feiler, M. S. Weiss, E. C. Schulz, P. Mehrabi, K. Karnicar, A. Usenik, J. Loboda, H. Tidow, A. Chari, R. Hilgenfeld, C. Uetrecht, R. Cox, A. Zaliani, T. Beck, M. Rarey, S. Gunther, D. Turk, W. Hinrichs, H. N. Chapman, A. R. Pearson, C. Betzel, A. Meents, X-ray screening identifies active site and allosteric inhibitors of SARS-CoV-2 main protease. *Science* **372**, 642–646 (2021).

33. E. A. MacDonald, G. Frey, M. N. Namchuk, S. C. Harrison, S. M. Hinshaw, I. W. Windsor, Recognition of divergent viral substrates by the SARS-CoV-2 main protease. *ACS Infect. Dis.* **7**, 2591–2595 (2021).

34. A. M. Shaqra, S. Zvornicanin, Q. Y. Huang, G. J. Lockbaum, M. Knapp, L. Tandeske, D. T. Barkan, J. Flynn, D. N. A. Bolon, S. Moquin, D. Dovala, N. Kurt Yilmaz, C. A. Schiffer, Defining the substrate envelope of SARS-CoV-2 main protease to predict and avoid drug resistance. *Nat. Commun.* **13**, 3556 (2022).

35. Y. Zhao, Y. Zhu, X. Liu, Z. Jin, Y. Duan, Q. Zhang, C. Wu, L. Feng, X. Du, J. Zhao, M. Shao, B. Zhang, X. Yang, L. Wu, X. Ji, L. W. Guddat, K. Yang, Z. Rao, H. Yang, Structural basis for replicase polyprotein cleavage and substrate specificity of main protease from SARS-CoV-2. *Proc. Natl. Acad. Sci. U.S.A.* **119**, e2117142119 (2022).

36. S. G. Sawicki, D. L. Sawicki, S. G. Siddell, A contemporary view of coronavirus transcription. *J. Virol.* **81**, 20–29 (2007).

37. V. V. Courouble, S. K. Dey, R. Yadav, J. Timm, J. Harrison, F. X. Ruiz, E. Arnold, P. R. Griffin, Revealing the structural plasticity of SARS-CoV-2 nsp7 and nsp8 using structural proteomics. *J. Am. Soc. Mass Spectrom.* **32**, 1618–1630 (2021).

38. D. M. Ferraro, N. Lazo, A. D. Robertson, EX1 hydrogen exchange and protein folding. *Biochemistry* **43**, 587–594 (2004).

39. C. B. Arrington, A. D. Robertson, Correlated motions in native proteins from MS analysis of NH exchange: Evidence for a manifold of unfolding reactions in ovomucoid third domain. *J. Mol. Biol.* **300**, 221–232 (2000).

40. J. Zheng, M. R. Chang, R. E. Stites, Y. Wang, J. B. Bruning, B. D. Pascal, S. J. Novick, R. D. Garcia-Ordonez, K. R. Stayrook, M. J. Chalmers, J. A. Dodge, P. R. Griffin, HDX reveals the conformational dynamics of DNA sequence specific VDR co-activator interactions. *Nat. Commun.* **8**, 923 (2017).

41. J. Zheng, C. Wang, M. R. Chang, S. C. Devarkar, B. Schweibenz, G. C. Crynen, R. D. Garcia-Ordonez, B. D. Pascal, S. J. Novick, S. S. Patel, J. Marcotrigiano, P. R. Griffin, HDX-MS reveals dysregulated checkpoints that compromise discrimination against self RNA during RIG-I mediated autoimmunity. *Nat. Commun.* **9**, 5366 (2018).

42. J. Y. Zhou, L. Y. Yang, A. DeColli, C. F. Meyers, N. S. Nemeria, F. Jordan, Conformational dynamics of 1-deoxy-D-xylulose 5-phosphate synthase on ligand binding revealed by H/D exchange MS. *Proc. Natl. Acad. Sci. U.S.A.* **114**, 9355–9360 (2017).

43. J. Y. Yang, Y. Zhang, I-TASSER server: New development for protein structure and function predictions. *Nucleic Acids Res.* **43**, W174–W181 (2015).

44. S. P. Meisburger, A. B. Taylor, C. A. Khan, S. N. Zhang, P. F. Fitzpatrick, N. Ando, Domain movements upon activation of phenylalanine hydroxylase characterized by crystallography

and chromatography-coupled small-angle x-ray scattering. *J. Am. Chem. Soc.* **138**, 6506–6516 (2016).

45. Y. J. Zhai, F. Sun, X. M. Li, H. Pang, X. L. Xu, M. Bartlam, Z. H. Rao, Insights into SARS-CoV transcription and replication from the structure of the nsp7-nsp8 hexadecamer. *Nat. Struct. Mol. Biol.* **12**, 980–986 (2005).

46. H. S. Hillen, Structure and function of SARS-CoV-2 polymerase. *Curr. Opin. Virol.* **48**, 82–90 (2021).

47. H. S. Hillen, G. Kokic, L. Farnung, C. Dienemann, D. Tegunov, P. Cramer, Structure of replicating SARS-CoV-2 polymerase. *Nature* **584**, 154–156 (2020).

48. B. Malone, E. A. Campbell, S. A. Darst, CoV-er all the bases: Structural perspectives of SARS-CoV-2 RNA synthesis. *Enzyme* **49**, 1–37 (2021).

49. R. T. Bradshaw, F. Marinelli, J. D. Faraldo-Gomez, L. R. Forrest, Interpretation of HDX data by maximum-entropy reweighting of simulated structural ensembles. *Biophys. J.* **118**, 1649–1664 (2020).

50. K. C. Kihn, T. Wilson, A. K. Smith, R. T. Bradshaw, P. L. Wintrode, L. R. Forrest, A. Wilks, D. J. Deredge, Modeling the native ensemble of PhuS using enhanced sampling MD and HDX-ensemble reweighting. *Biophys. J.* **120**, 5141–5157 (2021).

51. D. Franke, D. I. Svergun, *DAMMIF*, a program for rapid ab-initio shape determination in small-angle scattering. *J. Appl. Crystallogr.* **42**, 342–346 (2009).

52. D. I. Svergun, Restoring low resolution structure of biological macromolecules from solution scattering using simulated annealing. *Biophys. J.* **76**, 2879–2886 (1999).

53. Y. Hamuro, S. J. Coales, Hydrogen/deuterium exchange mass spectrometry for weak binders. *J. Am. Soc. Mass Spectrom.* **33**, 735–739 (2022).

54. A. J. Percy, M. Rey, K. M. Burns, D. C. Schriemer, Probing protein interactions with hydrogen/deuterium exchange and mass spectrometry-a review. *Anal. Chim. Acta* **721**, 7–21 (2012).

55. S. G. Sawicki, D. L. Sawicki, Coronavirus transcription: A perspective. *Curr. Top. Microbiol. Immunol.* **287**, 31–55 (2005).

56. A. T. Nguyen, C. L. Feasley, K. W. Jackson, T. J. Nitz, K. Salzwedel, G. M. Air, M. Sakalian, The prototype HIV-1 maturation inhibitor, bevirimat, binds to the CA-SP1 cleavage site in immature Gag particles. *Retrovirology* **8**, 101 (2011).

57. M. D. Purdy, D. Shi, J. Chrustowicz, J. Hattne, T. Gonen, M. Yeager, MicroED structures of HIV-1 Gag CTD-SP1 reveal binding interactions with the maturation inhibitor bevirimat. *Proc. Natl. Acad. Sci. U.S.A.* **115**, 13258–13263 (2018).

58. M. Z. Wang, C. M. Quinn, J. R. Perilla, H. L. Zhang, R. Shirra, G. J. Hou, I. J. Byeon, C. L. Suiter, S. Ablan, E. Urano, T. J. Nitz, C. Aiken, E. O. Freed, P. J. Zhang, K. Schulten, A. M. Gronenborn, T. Polenova, Quenching protein dynamics interferes with HIV capsid maturation. *Nat. Commun.* **8**, 1779 (2017).

59. R. E. London, HIV-1 reverse transcriptase: A metamorphic protein with three stable states. *Structure* **27**, 420–426 (2019).

60. A. F. Dishman, B. F. Volkman, Unfolding the mysteries of protein metamorphosis. *ACS Chem. Biol.* **13**, 1438–1446 (2018).

61. T. J. El-Baba, C. A. Lutomski, A. L. Kantsadi, T. R. Malla, T. John, V. Mikhailov, J. R. Bolla, C. J. Schofield, N. Zitzmann, I. Vakonakis, C. V. Robinson, Allosteric inhibition of the SARS-CoV-2 main protease: Insights from mass spectrometry based assays. *Angew. Chem. Int. Ed.* **59**, 23544–23548 (2020).

62. Z. C. Sun, L. Wang, X. Y. Li, C. P. Fan, J. F. Xu, Z. Z. Shi, H. R. Qiao, Z. Y. Lan, X. Zhang, L. Y. Li, X. Zhou, Y. Geng, An extended conformation of SARS-CoV-2 main protease reveals allosteric targets. *Proc. Natl. Acad. Sci. U.S.A.* **119**, e2120913119 (2022).

63. M. Gotte, Remdesivir for the treatment of Covid-19: The value of biochemical studies. *Curr. Opin. Virol.* **49**, 81–85 (2021).

64. M. A. Redhead, C. D. Owen, L. Brewitz, A. H. Collette, P. Lukacik, C. Strain-Damerell, S. W. Robinson, P. M. Collins, P. Schafer, M. Swindells, C. J. Radoux, I. N. Hopkins, D. Fearon, A. Douangamath, F. von Delft, T. R. Malla, L. Vangeel, T. Vercruysse, J. Thibaut, P. Leyssen, T. T. Nguyen, M. Hull, A. Tumber, D. J. Hallett, C. J. Schofield, D. I. Stuart, A. L. Hopkins, M. A. Walsh, Bispecific repurposed medicines targeting the viral and immunological arms of COVID-19. *Sci. Rep.* **11**, 13208 (2021).

65. N. Sluis-Cremer, G. Tachedjian, Mechanisms of inhibition of HIV replication by non-nucleoside reverse transcriptase inhibitors. *Virus Res.* **134**, 147–156 (2008).

66. X. Y. Xue, H. T. Yang, W. Shen, Q. Zhao, J. Li, K. L. Yang, C. Chen, Y. H. Jin, M. Bartlam, Z. H. Rao, Production of authentic SARS-CoV M-pro with enhanced activity: Application as a novel tag-cleavage endopeptidase for protein overproduction. *J. Mol. Biol.* **366**, 965–975 (2007).

67. D. R. Littler, B. S. Gully, R. N. Colson, J. Rossjohn, Crystal structure of the SARS-CoV-2 non-structural protein 9, nsp9. *iScience* **23**, 101258 (2020).

68. F. Liu, P. Lossl, R. Scheltema, R. Viner, A. J. R. Heck, Optimized fragmentation schemes and data analysis strategies for proteome-wide cross-link identification. *Nat. Commun.* **8**, 15473 (2017).

69. F. Liu, D. T. S. Rijkers, H. Post, A. J. R. Heck, Proteome-wide profiling of protein assemblies by cross-linking mass spectrometry. *Nat. Methods* **12**, 1179–1184 (2015).

70. B. MacLean, D. M. Tomazela, N. Shulman, M. Chambers, G. L. Finney, B. Frewen, R. Kern, D. L. Tabb, D. C. Liebler, M. J. MacCoss, Skyline: An open source document editor for creating and analyzing targeted proteomics experiments. *Bioinformatics* **26**, 966–968 (2010).

71. Z. Chen, J. Rappsilber, A generic solution for quantifying cross-linked peptides using software Skyline. *Protoc. Exch.* 10.1038/protex.2018.001 (2018).

72. Z. A. Chen, J. Rappsilber, Quantitative cross-linking/mass spectrometry to elucidate structural changes in proteins and their complexes. *Nat. Protoc.* **14**, 171–201 (2019).

73. M. Choi, C. Y. Chang, T. Clough, D. Broudy, T. Killeen, B. MacLean, O. Vitek, MSstats: An R package for statistical analysis of quantitative mass spectrometry-based proteomic experiments. *Bioinformatics* **30**, 2524–2526 (2014).

74. C. W. Combe, L. Fischer, J. Rappsilber, xiNET: Cross-link network maps with residue resolution. *Mol. Cell. Proteomics* **14**, 1137–1147 (2015).

75. Y. Perez-Riverol, A. Csordas, J. W. Bai, M. Bernal-Llinares, S. Hewapathirana, D. J. Kundu, A. Inuganti, J. Griss, G. Mayer, M. Eisenacher, E. Perez, J. Uszkoreit, J. Pfeuffer, T. Sachsenberg, S. Yilmaz, S. Tiwary, J. Cox, E. Audain, M. Walzer, A. F. Jarnuczak, T. Ternent, A. Brazma, J. A. Vizcaino, The PRIDE database and related tools and resources in 2019: Improving support for quantification data. *Nucleic Acids Res.* **47**, D442–D450 (2019).

76. M. J. Chalmers, S. A. Busby, B. D. Pascal, Y. J. He, C. L. Hendrickson, A. G. Marshall, P. R. Griffin, Probing protein ligand interactions by automated hydrogen/deuterium exchange mass spectrometry. *Anal. Chem.* **78**, 1005–1014 (2006).

77. Z. Zhang, D. L. Smith, Determination of amide hydrogen exchange by mass spectrometry: A new tool for protein structure elucidation. *Protein Sci.* **2**, 522–531 (1993).

78. B. D. Pascal, S. Willis, J. L. Lauer, R. R. Landgraf, G. M. West, D. Marciano, S. Novick, D. Goswami, M. J. Chalmers, P. R. Griffin, HDX workbench: Software for the analysis of H/D exchange MS data. *J. Am. Soc. Mass Spectrom.* **23**, 1512–1521 (2012).

79. T. R. Keppel, D. D. Weis, Mapping residual structure in intrinsically disordered proteins at residue resolution using millisecond hydrogen/deuterium exchange and residue averaging. *J. Am. Soc. Mass Spectrom.* **26**, 547–554 (2015).

80. N. Kirby, N. Cowieson, A. M. Hawley, S. T. Mudie, D. J. McGillivray, M. Kusel, V. Samardzic-Boban, T. M. Ryan, Improved radiation dose efficiency in solution SAXS using a sheath flow sample environment. *Acta Crystallogr. D Struct. Biol.* **72**, 1254–1266 (2016).

81. J. B. Hopkins, R. E. Gillilan, S. Skou, BioXTAS RAW: Improvements to a free open-source program for small-angle x-ray scattering data reduction and analysis. *J. Appl. Cryst.* **50**, 1545–1553 (2017).

82. V. V. Volkov, D. I. Svergun, Uniqueness of ab initio shape determination in small-angle scattering. *J. Appl. Cryst.* **36**, 860–864 (2003).

83. D. Franke, M. V. Petoukhov, P. V. Konarev, A. Panjkovich, A. Tuukkanen, H. D. T. Mertens, A. G. Kikhney, N. R. Hajizadeh, J. M. Franklin, C. M. Jeffries, D. I. Svergun, ATSAS 2.8: A comprehensive data analysis suite for small-angle scattering from macromolecular solutions. *J. Appl. Cryst.* **50**, 1212–1225 (2017).

84. L. Willard, A. Ranjan, H. Zhang, H. Monzavi, R. F. Boyko, B. D. Sykes, D. S. Wishart, VADAR: A web server for quantitative evaluation of protein structure quality. *Nucleic Acids Res.* **31**, 3316–3319 (2003).

85. A. Shevchenko, M. Wilm, O. Vorm, M. Mann, Mass spectrometric sequencing of proteins silver-stained polyacrylamide gels. *Anal. Chem.* **68**, 850–858 (1996).

86. D. E. Sleat, M. C. Della Valle, H. Zheng, D. F. Moore, P. Lobel, The mannose 6-phosphate glycoprotein proteome. *J. Proteome Res.* **7**, 3010–3021 (2008).

87. R. Craig, R. C. Beavis, TANDEM: Matching proteins with tandem mass spectra. *Bioinformatics* **20**, 1466–1467 (2004).

88. D. Franke, C. M. Jeffries, D. I. Svergun, Correlation map, a goodness-of-fit test for one-dimensional x-ray scattering spectra. *Nat. Methods* **12**, 419–422 (2015).

89. D. I. Svergun, Determination of the regularization parameter in indirect-transform methods using perceptual criteria. *J. Appl. Cryst.* **25**, 495–503 (1992).

90. R. P. Rambo, J. A. Tainer, Accurate assessment of mass, models and resolution by small-angle scattering. *Nature* **496**, 477–481 (2013).

91. V. Piiadov, E. Ares de Araujo, M. Oliveira Neto, A. F. Craievich, I. Polikarpov, SAXSMoW 2.0: Online calculator of the molecular weight of proteins in dilute solution from experimental SAXS data measured on a relative scale. *Protein Sci.* **28**, 454–463 (2019).
